# Folic acid–conjugated mesoporous silica particles as nanocarriers of natural prodrugs for cancer targeting and antioxidant action

**DOI:** 10.18632/oncotarget.25470

**Published:** 2018-05-29

**Authors:** Khaled AbouAitah, Anna Swiderska-Sroda, Ahmed A. Farghali, Jacek Wojnarowicz, Agata Stefanek, Stanislaw Gierlotka, Agnieszka Opalinska, Abdou K. Allayeh, Tomasz Ciach, Witold Lojkowski

**Affiliations:** ^1^ Department of Medicinal and Aromatic Plants Research, Pharmaceutical and Drug Industries Research Division, National Research Centre (NRC), Dokki, Giza, Egypt; ^2^ Laboratory of Nanostructures, Institute of High Pressure Physics, Polish Academy of Sciences, Warsaw, Poland; ^3^ Materials Science and Nanotechnology Department, Faculty of Postgraduate Studies for Advanced Sciences, Beni-Suef University, Beni-Suef, Egypt; ^4^ Biomedical Engineering Laboratory, Faculty of Chemical and Process Engineering, Warsaw University of Technology, Warsaw, Poland; ^5^ Environmental Virology Laboratory, National Research Centre (NRC), Dokki, Giza, Egypt

**Keywords:** mesoporous silica nanoparticles, hepatocellular carcinoma, targeted drug delivery, apoptosis, natural pro-drug

## Abstract

Naturally derived prodrugs have a wide range of pharmacological activities, including anticancer, antioxidant, and antiviral effects. However, significant barriers inhibit their use in medicine, e.g. their hydrophobicity. In this comprehensive study, we investigated simple and effective nanoformulations consisting of amine-functionalized and conjugated with folic acid (FA) mesoporous silica nanoparticles (MSNs). Two types of MSNs were studied: KCC- 1, with mean size 324 nm and mean pore diameter 3.4 nm, and MCM - 41, with mean size 197 and pore diameter 2 nm. Both types of MSNs were loaded with three anticancer prodrugs: curcumin, quercetin, and colchicine. The nanoformulations were tested to target *in vitro* human hepatocellular carcinoma cells (HepG2) and HeLa cancer cells. The amine-functionalized and FA-conjugated curcumin-loaded, especially KCC-1 MSNs penetrated all cells organs and steadily released curcumin. The FA-conjugated MSNs displayed higher cellular uptake, sustained intracellular release, and cytotoxicity effects in comparison to non-conjugated MSNs. The KCC-1 type MSNs carrying curcumin displayed the highest anticancer activity. Apoptosis was induced through specific signaling molecular pathways (caspase-3, H_2_O_2_, c-MET, and MCL-1). The nanoformulations displayed also an enhanced antioxidant activity compared to the pure forms of the prodrugs, and the effect depended on the time of release, type of MSN, prodrug, and assay used. FA-conjugated MSNs carrying curcumin and other safe natural prodrugs offer new possibilities for targeted cancer therapy.

## INTRODUCTION

Nanomedicine is defined as nanotechnology applications in medicine and is expected to provide tremendous opportunities for novel and effective strategies in the diagnosis and therapy of several diseases [1]. Nanomedicine has developed very rapidly in recent decades, yielding several commercial products [2–5]. One of the major goals of nanomedicine is to synthesize and tailor suitable drug delivery vehicles for targeted anticancer drug delivery. Such targeted drugs may efficiently cross physiological barriers, accumulate in desirable sites, and sustainably release drugs for treatment of diseases and offer reduced negative side effects [6]. Numerous materials have been investigated in nanomedicine as drug delivery systems (DDSs), such as organic DDS-based liposomes, solid lipid nanoparticles, dendrimers, and polymeric micelles. However, several limitations are associated with such organic-based carriers in DDSs, including their insufficient stability [7] and not fully controlled release mechanisms [8]. On the other hand, inorganic DDS-based nanoparticles, including magnetic nanoparticles, gold nanoparticles, carbon nanotubes, silica nanoparticles, and many other inorganic nanomaterials, have considerable potential as promising alternatives to organic systems in biomedical applications [6]. Among the inorganic DDSs, mesoporous silica nanoparticles (MSNs) offer many advantages such as physicochemical and biochemical stability and good biocompatibility [9–11]. Silica nanoparticles had been approved by the U.S. Food and Drug Administration (FDA) for first time in cancer imaging in clinical trials [12]. Thus, there is a high potential for silica nanoparticles as DDSs for clinical applications [13].

Attention to MSNs has recently increased because of their unique properties, including straightforward synthesis, high surface area, possibility of pore structure optimization with pore size ranging from 2 to 50 nm, facile functionalization of their surfaces by functional groups, good biocompatibility, excellent endocytotic behavior, and low-toxicity in biological systems [10, 14, 15].

Cellular uptake is considered a key success factor to increase the antitumor efficiency of drugs. Among the most promising applicable strategies is cancer cells targeting by using molecular ligands that are specifically overexpressed in cancerous tissues. Especially, receptor-mediated endocytosis depends on specific ligands that can distinguish specific receptors found in cell membranes [16]. Among the various targeting molecule’s ligands (e.g., mannose, hyaluronic acid, arginine–glycine aspartate, lactobionic acid), folic acid (FA) is a small molecule commonly known as vitamin B9. FA is important in nucleic acid production, cell division, and metabolic processes for cells [17, 18]. The folate receptor is useful for cancer targeting because of its overexpression in many human cancers including ovarian, kidney, breast, myeloid cells, and brain and lung cancer cells. Moreover, folate receptor density increases when the cancer worsens [18]. Owing to these FA properties, many research groups have employed FA to develop cancer-targeting systems based on MSNs [19–21].

Among cancers, hepatocellular carcinoma (HCC) is a common primary liver malignancy and a major cause of cancer-related death worldwide [22]. Several chemotherapeutic agents approved by the FDA for clinical use, such as sorafenib, improve survival rates and time to radiologic progression in HCC treatments [23]. However, the results of using several anticancer drugs for treating HCC are limited because of strong drug resistance [24]. Therefore, novel therapeutic formulations to enhance efficacy and target drugs are urgently needed.

Cancers can be targeted by different molecular pathways, and the apoptotic pathways (either intrinsic or extrinsic) have been considered as promising [25, 26]. Apoptosis is a process by which cells undergo programmed cell death [25]. Several signaling pathways induce apoptosis in cancer cells. Of special interest for the present study are the following signaling pathways: activation of caspase-3 [27]; inhibition of the anti-apoptotic Bcl-2 family, such as myeloid cell leukemia 1 (Mcl-1) [28]; suppression of mesenchymal–epithelial transition factor (c-MET), a receptor tyrosine kinase that binds hepatocyte growth factor (HGF) [29]; and the intracellular overproduction of hydrogen peroxide (H_2_O_2_) in mitochondria [30].

Recently, use of natural anticancer products obtained from plants has attracted much attention [31–33]. Some of these products have shown high therapeutic efficacy in preclinical investigations and exhibit better safety profiles than synthetic drugs. Especially, the antioxidant properties of natural substances offer a significant promise for reducing the risk for diseases such as cancer, fibrosis, liver injury, diabetes, aging, and others related to oxidative stress and reactive oxygen species (ROS). However, natural anticancer drugs are mostly hydrophobic, resulting in low aqueous solubility and thus low bioavailability, so that concentrations in the desired tissue are insufficient.

Nanotechnology-based strategies for targeted drug delivery are being developed to overcome above barriers [34–36]. The principle of this strategy is to connect the hydrophobic anticancer drug with a hydrophilic carrier that can cross barriers to cancer cells and become internalized by cells.

In this study, our attention was focused on three natural anticancer drugs: curcumin (CR), quercetin (QR), and colchicine (COL) [37–42]. In our previous reports, we demonstrated controlled release of CR and QR loaded to two types of MSNs (KCC-1 and MCM-41) [43, 44]. The results demonstrated that especially the KCC-1 type has great promise for controlling the release of CR and QR. In the current study, we further explored the potential application of the above two MSNs types, conjugated with FA ligands, and loaded with CR and QR, as well as a COL, for antioxidant properties and anticancer activity. The present study may provide useful information for selecting natural prodrugs for further development based on promising selective anticancer activity. To our knowledge, no such comprehensive comparisons have been reported based on a delivery system using MSNs.

## RESULTS

Two types of MSNs (KCC-1 and MCM-41) were studied as pro-drug carriers in the present study. In the following text, the following nomenclature will be used:

As-synthesized MSNs: MCM-Calcined and KCC-Calcined.

NH_2_ functionalized MSNs: KCC-NH_2_ and MCM-NH_2._

FA conjugated MSNs: KCC-NH_2_-FA and MCM-NH_2_-FA.

Drug loaded amine-modified MSNs: MCM-NH_2_-CR/QR/COL, and KCC-NH_2_-CR/QR/COL.

Drug loaded FA-conjugated MSNs: KCC-NH_2_-FA-CR/QR/Col, and MCM-NH_2_-FA-CR/QR/Col, where CR stands for curcumin, QR for quercetin, and Col for colchicine.

Dye fluorescence–labeled MSNs: MCM-FITC and KCC-FITC.

### MSNs meso- and nanostructure

**Figure 1:**
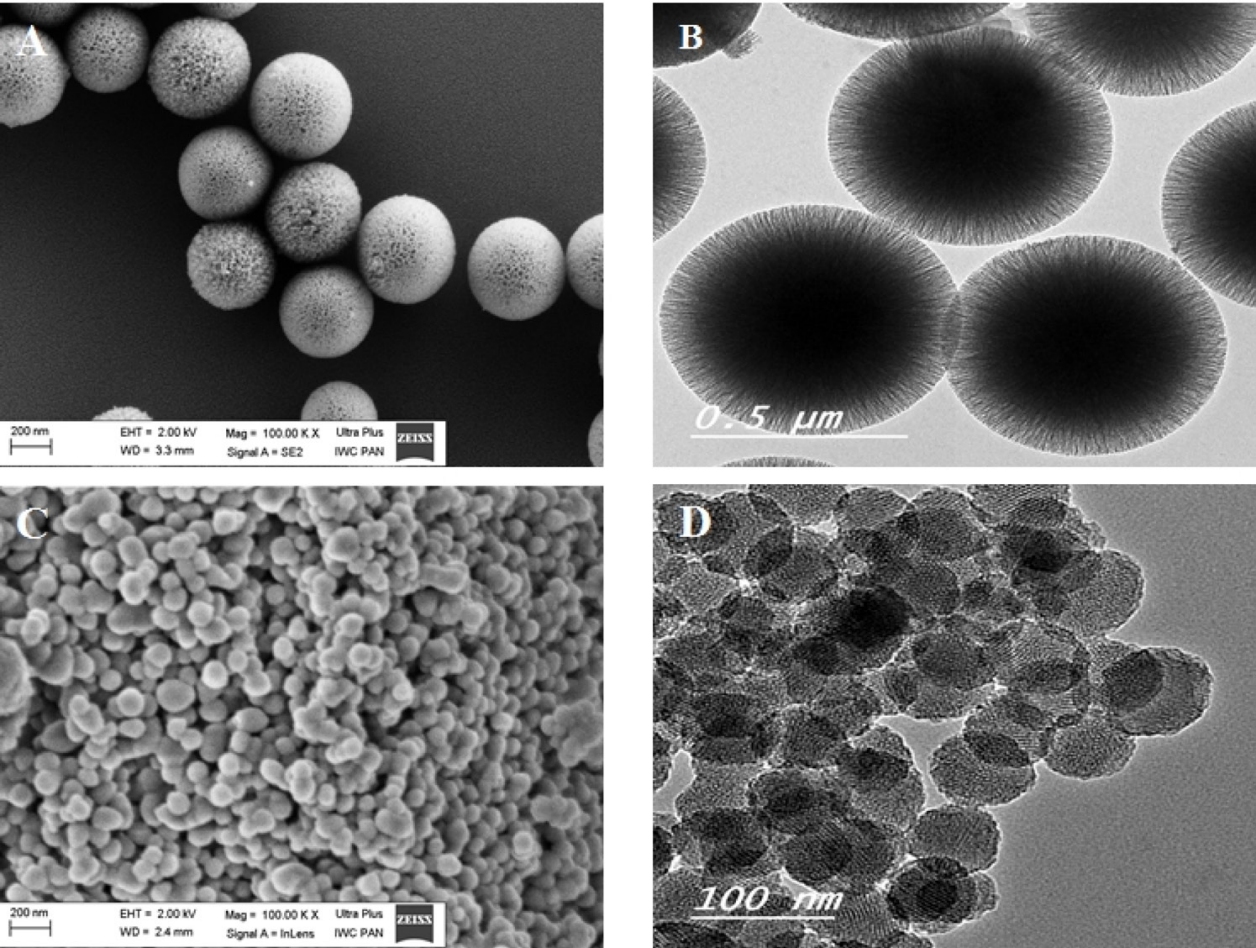
SEM (**A**, **C**) and TEM (**B**, **D**) images of mesoporous silica nanomaterials: KCC-Calcined (A, B) and MCM-Calcined (C, D).

The as-synthesized MSNs and their morphological characteristics are displayed in Figure 1 and in Supplementary Table 1. Scanning electron microscopy (SEM) and transmission electron microscopy (TEM) images (Figure 1A, 1B) show that KCC-Calcined MSNs take the form of monodisperse spherical nanoparticles with fibrous dendritic structure, with mean size 324 ± 33 and 3.4 nm pore diameter. The MCM-Calcined MSN was composed of spherical nanoparticles with mean size 197 ± 17 nm and nanopore diameter approximately 2 nm (Figure 1C, 1D). The MCM MSNs show a tendency for agglomeration comparing to the KCC ones. The results are in agreement with data given in ref [43, 44]. Detailed information on N_2_ adsorption/desorption measurements are given in Supplementary Table 1 in supplementary information.

### Drug loading characterization

The amount of FA molecules attached to the MSNs surface, and drug loaded into MSNs pores were measured by means of thermogravimetry (TG) and differential scanning calorimetry (DSC). The results are shown in supplementary material (Supplementary Table 1). The amount of FA molecules attached to MCM was lower than for the KCC; for MCM-NH_2_-FA, it was 2.5 wt.%, and for KCC-NH_2_-FA, it was 3.9 wt.%. For MCM MSNs, drug loading was as follows: 29.3% wt% for MCM-NH_2_-FA-CR, 31.6 wt% for MCM-NH_2_-FA-QR, and 2.9% wt.% for MCM-NH_2_-FA-COL. For KCC MSNs, drug loading was as follows: 19.5 wt% for KCC-NH_2_-FA-CR, 20.3 wt%, for KCC-NH_2_-FA-QR, and 2.3 wt% for KCC-NH_2_-FA-COL, respectively (Supplementary Figure 1 and Supplementary Table 1). The drug loading capacity of MCM-NH_2_-FA was slightly higher than that of KCC-NH_2_-FA, and drug loading with COL was significantly less than for CR and QR.

**Figure 2:**
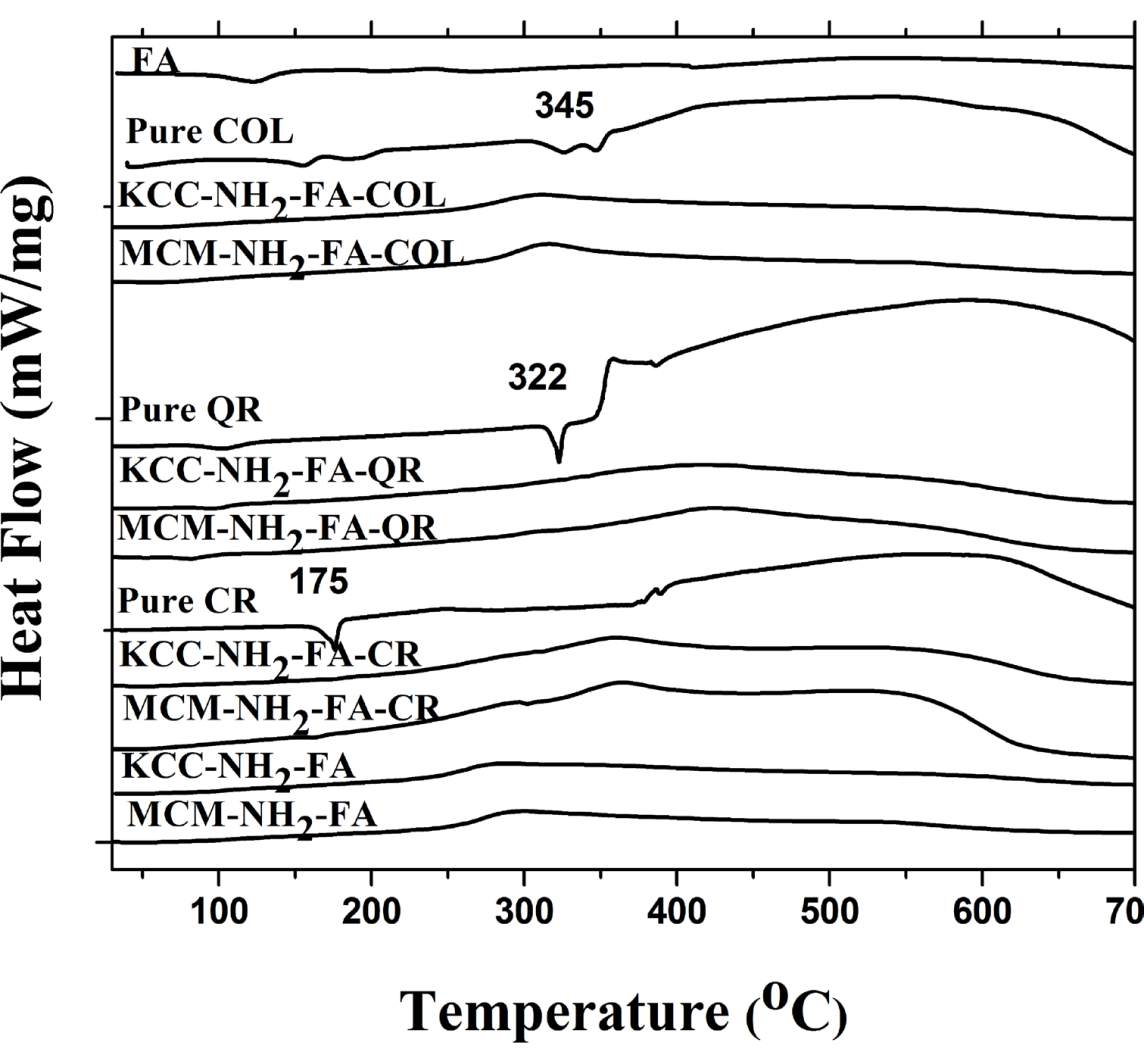
DSC profiles of as-synthesized (calcined) MSNs, FITC-labeled MSNs, FA-conjugated MSNs, drug-loaded FA-conjugated MSNs, and pure prodrugs

DSC studies confirmed efficient drug loading into the MSNs. We expect pro-drugs enclosed in the nanopores to take an amorphous form because of close contact with the amorphous silica matrix. Pure drugs displayed a single endothermic melting peak at 175° C, 322° C, and 345° C, characteristic for crystalline CR, QR, and COL. For comparison, DSC analysis of the prodrug-loaded MSNs (Figure 2) did not show any peaks at melting temperatures corresponding to pure drugs, indicating that the prodrugs were loaded into nanopores of the FA-conjugated MSNs, and as an amorphous material undergoes smooth glass transitions instead of crystallization.

**Figure 3:**
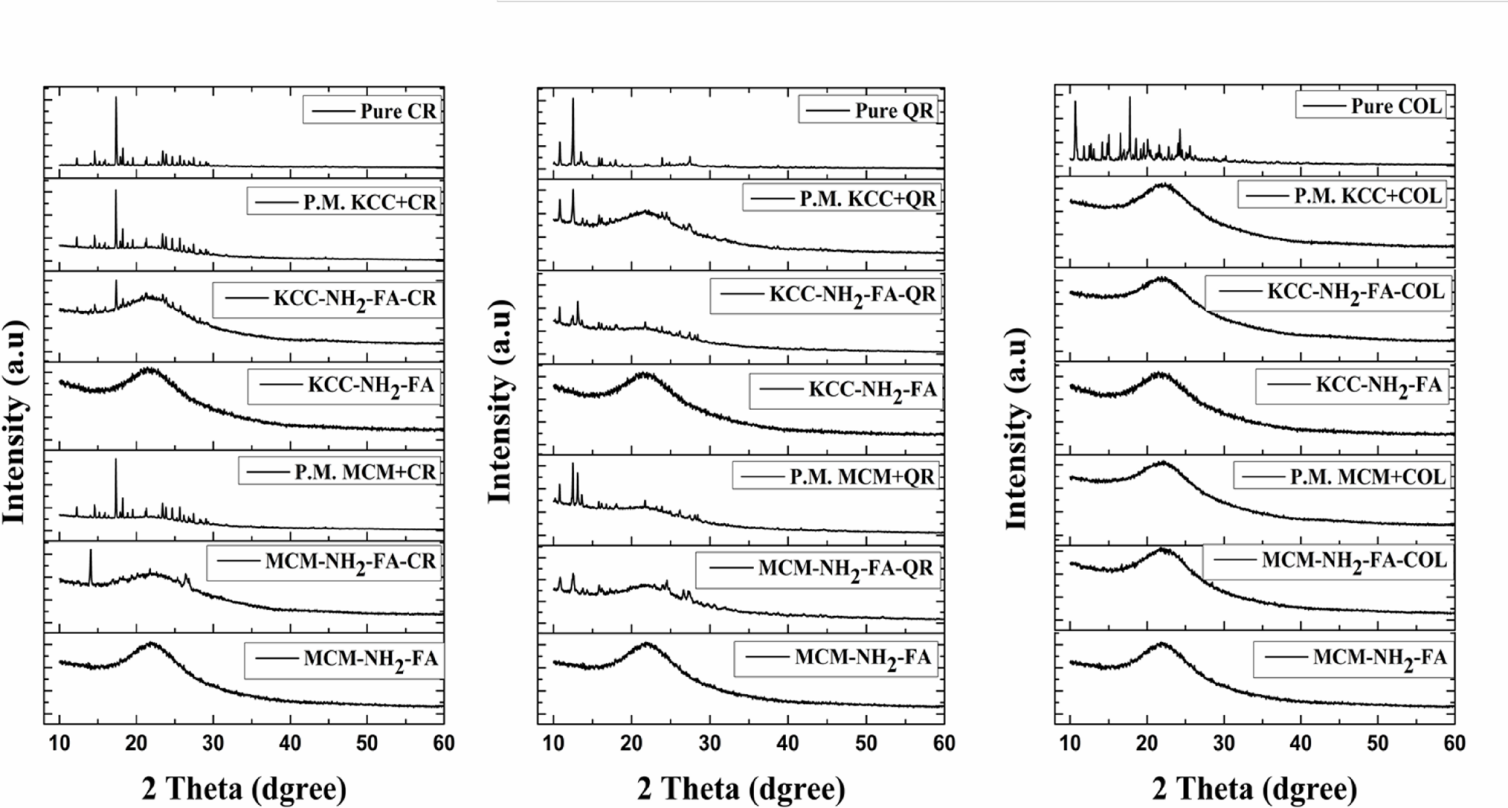
XRD patterns of FA-conjugated MSNs, drug-loaded FA-conjugated MSNs, and physical mixtures (P.M.) materials (simple mixing of drug with MSNs)

XRD patterns permit to estimate directly whether the pro drugs take a crystalline or amorphous form. XRD patterns of pure prodrugs and of MSNs loaded with drugs are shown in Figure 3. We also made a physical mixture of prodrugs and MSNs, to record a superposition of the XRD peaks and compare them with a spectrum for drug-loaded MSNs. The XRD patterns of the pure anticancer drugs present several peaks in the region from 10° to 30° of the diffraction pattern, corresponding to the crystalline phase of the prodrugs. For the physical mixture, several peaks with high intensities were obtained. In case of CR loaded MSNs, it is seen that there are small peaks corresponding to pure CR at 14.52° (MCM-NH_2_-FA-CR); 17.37° and 14.4° (KCC-FA-CR). In case of QR loaded MSNs, several peaks corresponding to pure QR at 10.68°, 12.54°, and 27.52° (MCM-NH_2_-FA-QR); and 10.68°, 12.54°, and 13.16° (KCC-NH_2_-FA-QR) were detected. However, the detected peaks in QR loaded MSNs were of small intensity compared with the prepared physical mixtures. In case of Col, no peaks were detected for COL loaded to both MSNs.

**Figure 4:**
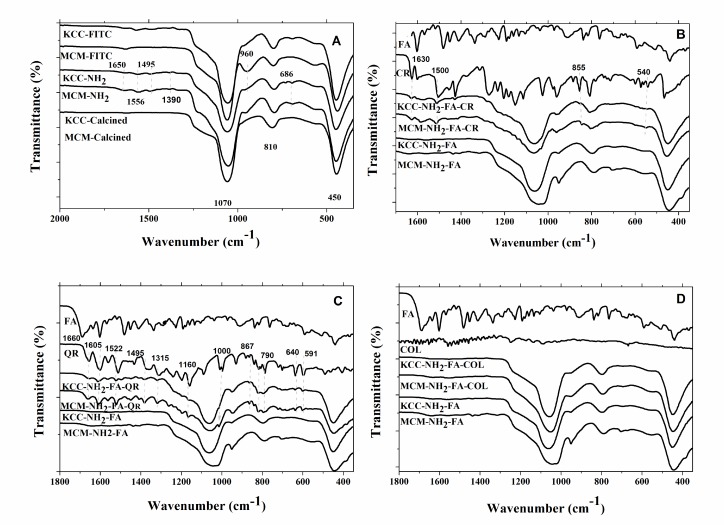
FT-IR spectra of: (**A**) as-synthesized (calcined) MSNs, amino-modified MSNs, and FITC-labeled MSNs (**B**); CR-loaded to FA-conjugated MSNs (B); QR-loaded FA-conjugated MSNs (**C**); and COL-loaded FA-conjugated MSNs COL-loaded to FA-conjugated MSNs (**D**).

Fourier transform-infrared (FT-IR) spectroscopy permits to identify the dominant chemical bonds between pro-drugs, molecules used for MSNs functionalization and silica mesospheres. The results for the Fourier transform-infrared (FT-IR) spectra are shown in Figure 4 as well as in supplementary information. The effect of drug-loading on the FT-IR spectra is displayed in Figure 4B–4D. In case of CR-loaded FA-conjugated MCM-NH_2_-FA-CR and KCC-NH_2_-FA-CR, bands at 1630 cm^-1^, 1500 cm^-1^, 855 cm^-1^, and 540 cm^-1^ were observed, corresponding to the pure CR spectrum (Figure 4B). In case of FT-IR spectra of QR-loaded FA-conjugated MSNs, several new bands at 1660 cm^-1^, 1605 cm^-1^, 1522 cm^-1^, 1495 cm^-1^, 1315 cm^-1^, 640 cm^-1^, and 591 cm^-1^ were observed corresponding to the pure QR spectrum (Figure 4C). In FT-IR spectra of COL-loaded materials, no bands were detected (Figure 4D), may be due to small loading percent compared other prodrugs. Thus, the FT-IR results confirmed successful surface modification, FA-conjugation, and drug loading to MSNs.

**Figure 5:**
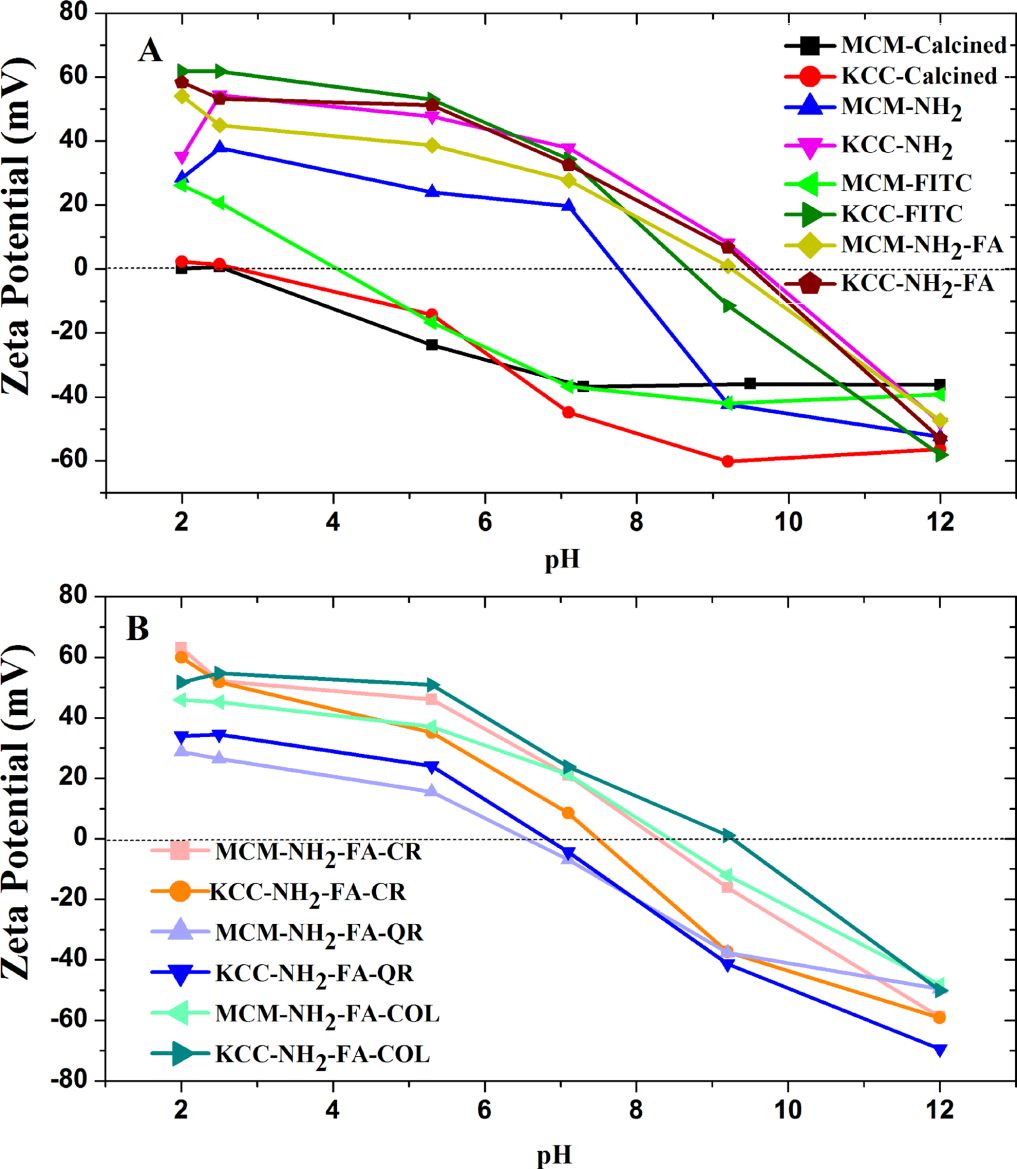
Zeta potential measurements of: (**A**) as-synthesized (calcined) MSNs, amino-functionalized MSNs, FITC-labeled MSNs, and FA-conjugated MSNs (**B**) prodrug-loaded FA-conjugated MSNs.

The zeta potential of all MSNs suspended in aqueous media was measured at a pH range from 2 to 12 and the results are shown in Figure 5. The zeta potential tells about the surface charge of the MSNs and their potential electrostatic attraction to cancer cells. Comprehensive results of zeta potential studies are described in supplementary information.

The calcined MSNs show a negative value of the zeta potential practically for the whole pH range, except for pH = 2. Also the zeta potential of the MCM-FITC sample rapidly decreased to negative values (Figure 5A). The FA conjugated MSNs acquired a negative charge at pH 9.5–10.0, only, similarly as the KCC-NH_2_ samples. The same samples displayed the higher zeta potential close to 60 mV in highly acidic environment of pH 2–2.5. The MCM-NH_2_ sample deteriorated to negative zeta potential values quicker than the KCC-NH_2_ sample, e.g., at pH = 8.

Figure 5B shows the zeta potential of FA-conjugated MSNs with prodrugs loaded, as a function of pH and type of prodrug. The MCM-NH_2_-FA and KCC-NH_2_-FA samples permit to assess the effect of drug loading on the zeta potential. The maximum values of the zeta potential at high acidity of pH = 2.0–2.5 are for both the drug loaded MCM-NH_2_-FA-CR and KCC-NH_2_-FA-CR and non-loaded samples close to 60 mV. However, CR loading accelerated the decrease of the zeta potential as pH is increased. The zero mV value is crossed in the pH range 8–9 comparing to 10 for the non-loaded ones. The crossing point was at highest pH of 9.5 for KCC-NH_2_-FA-COL followed by MCM-NH_2_-FA-CR, MCM-NH_2_-FA-COL. KCC-NH_2_-FA-CR, and QR loaded samples, with crossing point around pH 7. The difference in the crossing point between samples behaviour depending on loaded pro drug are in the range from pH 7 to pH 10.

Internalization of nanoparticles by cancer cells depends on their surface charges. Cancer environment is acidic and cancer cells tend to have a negative charge. Since all the drugs loaded MSNs display a positive zeta potential in acidic environment, they can be expected to be an efficient DDSs.

### Cellular uptake as a function of surface modification of nanoparticles and incubation time

**Figure 6:**
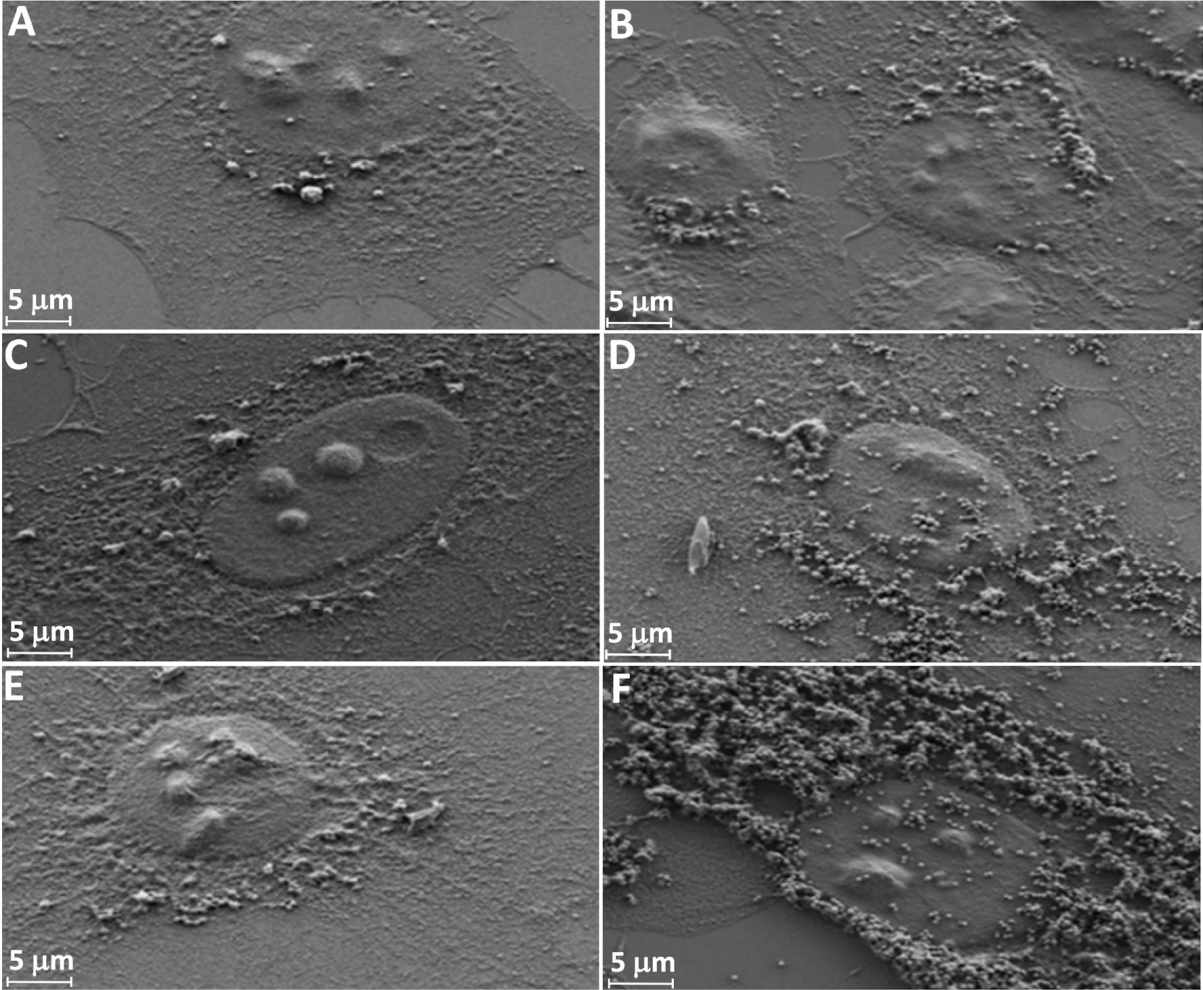
SEM images of cellular uptake of HeLa cells as a function of surface modification of MSNs after 24-h incubation time MCM-Calcined (**A**), KCC-Calcined (**B**), MCM-NH_2_ (C), KCC-NH_2_ (**D**), MCM-NH_2_-FA (**E**), and KCC-NH_2_-FA (**F**).

Internalization of MSNs in HeLa cells is shown by means of SEM in Figure 6. All FA-conjugated MSNs showed high internalization in HeLa cells, followed by amine-modified MSNs and as-synthesized ones (Figure 6). In addition, KCC-MSNs were well-distributed and dispersed in HeLa cells, mainly in cytoplasm and some in the nucleus (Figure 6B, 6D, 6F). In case of MCM-MSNs they were aggregated mainly in cytoplasm and some in the nucleus (Figure 6A, 6C, 6E).

**Figure 7:**
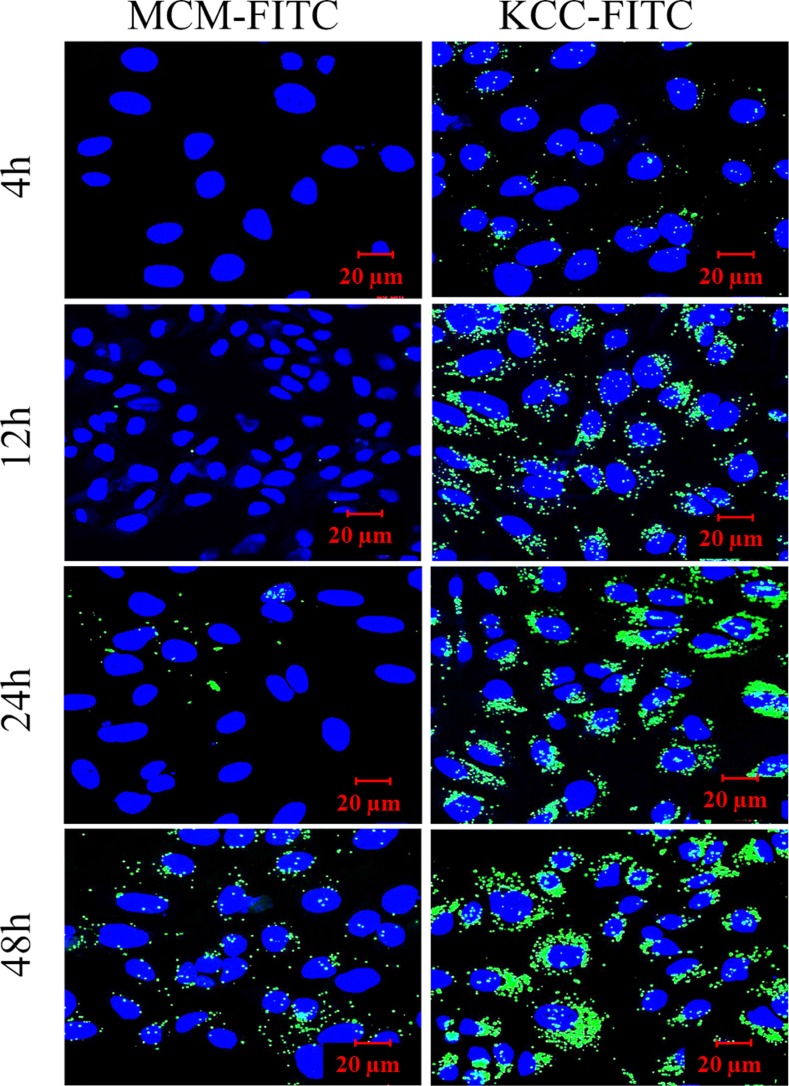
Confocal laser scanning microscopy (CLSM) images of cellular uptake of HeLa cells treated with FITC (green fluorescence) dye-labeled MSNs (MCM-FITC and KCC-FITC) as a function of time The cell nucleus was stained with DAPI (blue).

The effect of exposure time (4, 12, 24, and 48 h) on cellular uptake is shown in Figure 7. We used dye fluorescent–labeled MSNs with FITC dye for confocal laser scanning microscopy CLSM measurements. This is a frequently employed as model molecule in such studies. Green fluorescence was present in HeLa cells for both types of MSNs. Signal from both MCM-FITC and KCC-FITC MSNs gradually increased with time of incubation from 4 h to 48 h. It is seen that comparing to MCM-FITC MSNs, KCC-FITC are strongly attracted to the cells, surround them and become internalized. This is in agreement with the positive zeta potential of KCC-FITC, while the MCM -FITC ones have a negative zeta potential value.

### Intracellular release studies by means of CLSM

**Figure 8:**
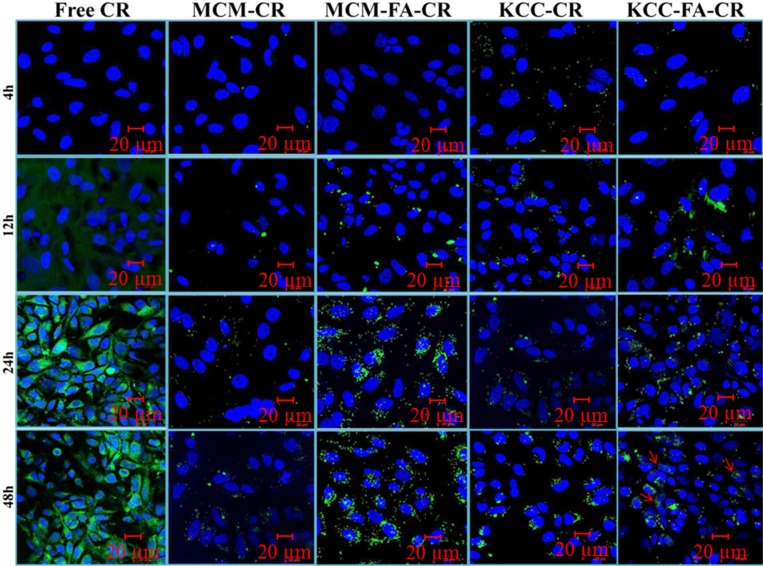
Confocal laser scanning microscopy (CLSM) images of intracellular and drug release in HeLa cells of different forms of CR (green fluorescence) as an anticancer prodrug as a function of time Vertical columns show free form, CR-loaded amino-functionalized MSNs (MCM-CR = denotes MCM-NH_2_-CR and KCC-CR = denotes KCC-NH_2_-CR), and CR-loaded FA-conjugated MSNs (MCM-FA-CR = denotes MCM-NH_2_-FA-CR and KCC-FA-CR = denotes KCC-NH_2_-FA-CR). The cell nucleus was stained with DAPI (blue). The release of CR prodrug can be seen by red arrows. Arrows indicate CR release after MSNs internalization.

Intracellular release studies were carried out for the CR pro-drug only. As shown in Figure 8, for pure CR, green fluorescence within HeLa cells was observed after 24 h incubation. An intensive green fluorescence from pure CR diffused inside cells was observed after 24 h and 48 h. Four formulations: MCM-NH_2_-CR, MCM-NH_2_-FA-CR, KCC-NH_2_-CR, and KCC-NH_2_-FA-CR were studied to explore the differences in intracellular release depending on the presence of FA ligands and MSN type. After 4 h and 12 h, for MCM-NH_2_-CR and MCM-NH_2_-FA-CR, and KCC-NH_2_-CR, almost no green florescence signal was detected within cells, compared to KCC-NH_2_-FA-CR. After further incubation for 48 h, a more green fluorescence corresponding to internalization and release CR was observed for both MSNs types (indicated by red arrows). However, for all nanoformulations, green signal fluorescence was located mainly in the perinuclear region, within cytoplasm, and only some in the nucleus (blue color) in HeLa cells. Remarkable enhancement in internalization and release of CR-loaded MSNs was observed for all FA-conjugated and CR-loaded MSNs compared to formulations without FA targeting molecules.

These results demonstrate that intracellular release properties increase with time of incubation, are improved by FA conjugation.

### *In vitro* cytotoxicity studies

**Figure 9:**
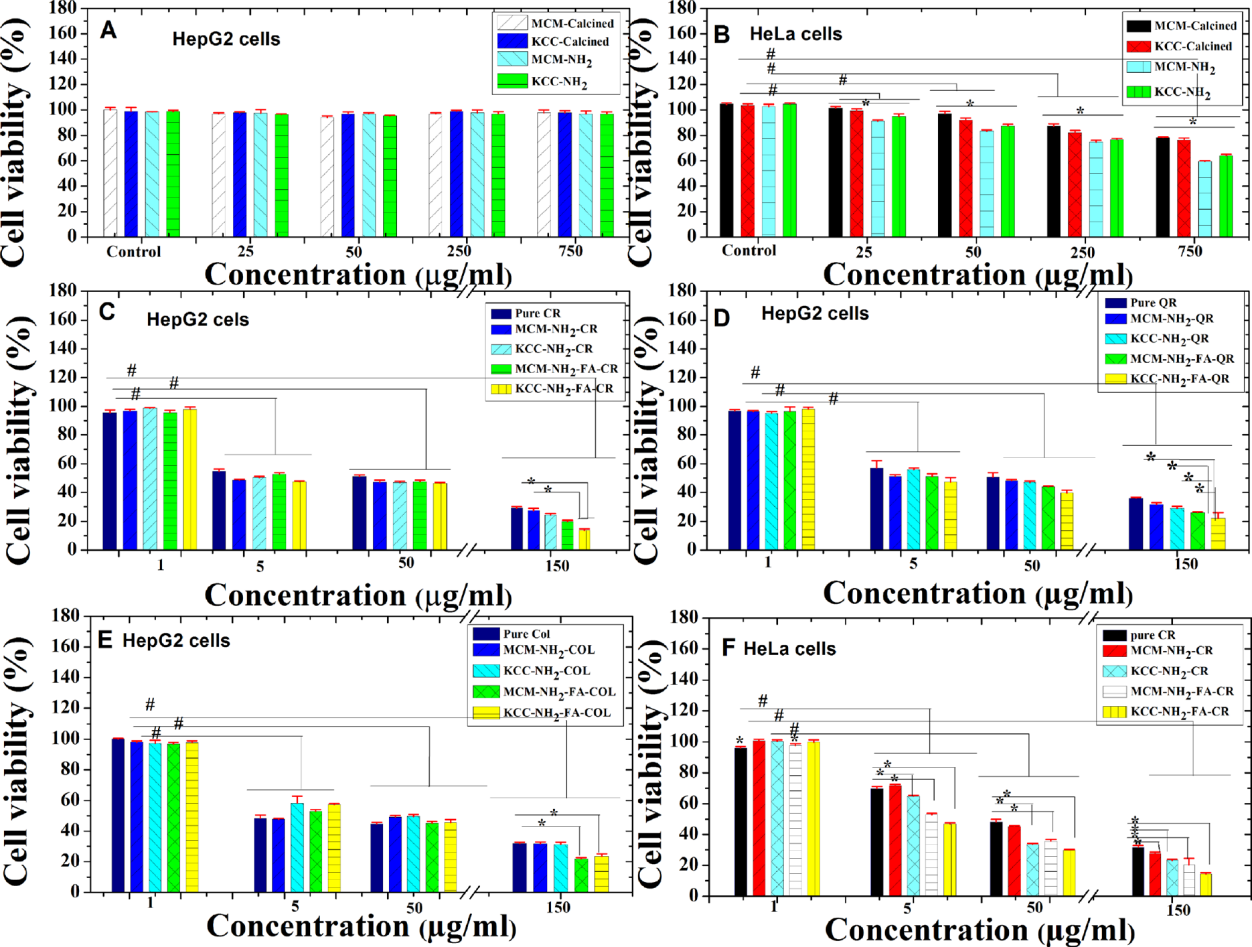
*In vitro* cytotoxicity and biocompatibility evaluation of anticancer natural prodrugs suspended in PBS buffer against HepG2 and HeLa cancer cells for 24 h of incubation (**A**) Biocompatibility of calcined MSNs and amino-functionalized MSNs with concentrations up to 750 µg/ml in HepG2 cells; (**B**) Biocompatibility of calcined MSNs and amino-functionalized MSNs with concentrations up to 750 µg/ml in HeLa cells; (**C**) cytotoxicity of pure CR and its nanoformulations in HepG2 cells; (**D**) cytotoxicity of QR and its nanoformulations in HepG2 cells; (**E**) cytotoxicity of COL and its nanoformulations; and (**F**) cytotoxicity of pure CR and its nanoformulations in HeLa cells. Notes: Data are expressed as mean and error bars represent ± standard deviation; ^*^p < 0.05 compared to samples under same concentration; ^#^*p* < 0.05 compared to concentration of 1 µg/ml (as control).

We tested the cytotoxicity of MSNs prior to FA conjugation and drug loading on two cancer cell lines (HepG2 and HeLa). Both calcined MSNs and amino-modified ones showed negligible cytotoxicities towards HepG2 cells, even at a high concentration of 750 µg/ml, and their viability remained high (above 95%) (Figure 9A). In case of HeLa cells, both MSNs showed significant cytotoxicity (*p* ˂ 0.05) effect, this depends on concentration (Figure 9B). The cell viability for a 750 μg/ml dose was as 59.5 ± 0.3% for MCM-NH_2_ and 63.8 ± 1.2% for KCC-NH_2_. The amino-modified MSNs showed slightly higher cytotoxicity compared to as-synthesized MSNs.

To confirm the role of FA in anticancer activity, we compared the cytotoxicity of prodrug-loaded MCM-NH_2_-FA and KCC-NH_2_-FA, with pure prodrug, and amino fuctionalized only: MCM-NH_2_ and KCC-NH_2_ (Figure 9C–9F). For 150 µg/ml concentration, and HepG2 cells, the following results were obtained. In the case of curcumin (Figure 9C), KCC-NH_2_-FA-CR strongly (*p* ˂ 0.05) inhibited cell viability (13.7 ± 0.8%) compared to MCM-NH_2_-CR (27.4 ± 1.3%) and pure CR (29.1 ± 1.2%). In the case of quercetin (Figure 9D), KCC-NH_2_-FA-QR significantly (*p* ˂ 0.05) decreased cell viability (22.1 ± 3.9%) compared to KCC-NH_2_-CR (28.9 ± 0.5%), MCM-NH_2_-FA-QR (26.1 ± 0.5), MCM-QR (34.7 ± 1.1%), and pure QR (34.2 ± 0.4%). In the case of COL-loaded MSNs (Figure 9E), FA conjugation also decreased cells viability: 23.5 ± 1.6% for KCC-NH_2_-FA-COL and 21.8 ± 0.8 % for MCM-NH_2_-FA-COL, both with a significantly (*p* ˂ 0.05) stronger effect than for pure COL (31.7 ± 0.4%), and amino functionalized ones: MCM-NH_2_-COL (31.7 ± 1.0%), and KCC-NH_2_-COL (31.1 ± 1.5%). Thus FA conjugated MSNs are most efficient in decreasing HepG2 cells viability, the amino acid functionalized ones are second in efficiency and pure pro drugs are least efficient.

To evaluate the anticancer effect on HeLa cells, we used only the CR prodrug (as the most effective prodrug for HepG2 cells) and the results are shown in Figure 9F. A significant decrease in cell viability as concentration is increased can be observed. For all CR loaded MSNs except MCM-NH_2_-CR, cells viability was significantly decreased comparing to pure CR. On the example of KCC type MSNs, for concentrations equivalent to 150 µg/ml, KCC-NH_2_-FA-CR strongly (*p* ˂ 0.05) decreased cell viability (14.4 ± 0.8%) compared to KCC-NH_2_-CR (23.3 ± 0.7%) and pure CR (31.5 ± 1.1%). It seems that the anticancer activity for pure CR prodrug and CR loaded into MSNs is the same in HepG2 and HeLa cells. The KCC-MSNs were more effective than MCM-MSNs when the prodrugs CR and QR are concerned. The KCC-NH_2_-FA-CR showed the highest cytotoxicity of all samples.

From the cytotoxicity results, we calculated IC50-the inhibition concentration for killing of 50% of cells (Table 1). A decreasing value of IC50 indicates a high anticancer activity. IC50 was reduced for prodrug-loaded FA-conjugated MSNs compared to prodrug-s loaded amine-functionalized MSNs and pure prodrugs. In the case of CR, the IC50 values were 28.9 ± 2.7 µg/ml for the pure prodrug, 23.8 ± 1.8 µg/ml for MCM-NH_2_-CR, 22.0 ± 0.4 µg/ml for MCM-NH_2_-FA-CR, 20.7 ± 1.2 µg/ml for KCC-NH_2_-CR, and 15.6 ± 0.5 µg/ml for KCC-NH_2_-FA-CR, respectively. The pure prodrug was least efficient, amine-functionalized MSNs loaded with CR ranked second, and FA-conjugated MSNs loaded with CR showed the strongest effect. At the same time, MCM MSNs always showed a smaller efficiency than KCC MSNs. The same trend was also observed for QR and COL.

**Table 1:** IC50 values (μg/ml) of CR, QR, COL, and their prepared nanoformulations after treating HepG2 cells for 24 h

Prodrug	Form and type of nanoparticle (NP)-loaded compounds				
	Pure	Amino-modified NPs		FA-modified NPs	
CR	***Pure CR***	***MCM-NH***_***2***_***-CR***	***KCC-NH***_***2***_***-CR***	***MCM-NH***_***2***_***-FA-CR***	***KCC-NH***_***2***_***-FA-CR***
	28.9 ± 2.7	23.8 ± 1.8	20.7 ± 1.2	22.0 ± 0.4	15.6 ± 0.5
QR	***Pure QR***	***MCM-NH***_***2***_***-QR***	***KCC-NH2-QR***	***MCM-*** ***NH***_***2***_***-FA-QR***	***KCC-NH***_***2***_***-FA-QR***
	35.2 ± 7.4	29.2 ± 0.4	35.2 ± 1.1	20.5 ± 1.2	16.8 ± 0.5
COL	***Pure COL***	***MCM-NH***_***2***_***-COL***	***KCC-NH***_***2***_***-COL***	***MCM-*** ***NH***_***2***_***-FA-COL***	***KCC-NH***_***2***_***-FA-COL***
	23.0 ± 0.7	19.8 ± 0.2	35.0 ± 4.5	19.1 ± 0.2	22.6 ± 0.8

Data calculated are expressed as mean ± standard deviation.

When efficiency of particular prodrugs was compared, the lowest IC50 value was obtained for KCC-NH_2_-FA-CR (15.6 ± 0.5 μg/ml), followed by KCC-NH_2_-FA-QR (16.8 ± 0.5 µg/ml), and KCC-NH_2_-FA-COL (22.6 ± 0.8 µg/ml). Based on the IC50 quantification, CR was selected for further exploration of the anticancer mechanism using several assays.

### Activation of caspase-3 pathway in HepG2 cells by the CR nanoformulation

**Figure 10:**
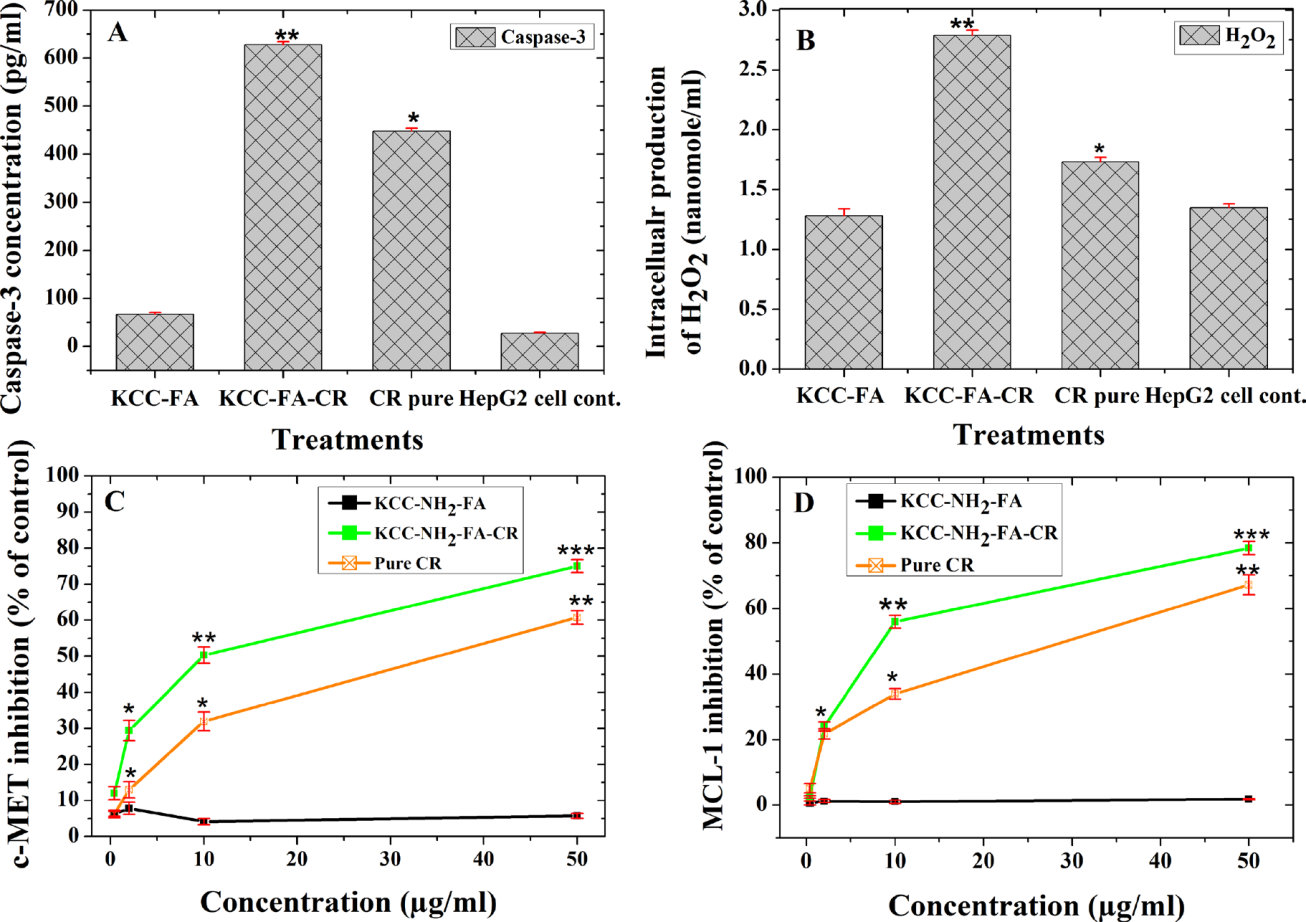
Molecular signaling pathways of CR-loaded FA-conjugated KCC-1 MSN (KCC-NH_2_-FA-CR) nanoformulation, pure CR, and KCC-NH_2_-FA MSNs on human HCC cells (HepG2) (**A**) caspase-3 activation for apoptosis pathway; (**B**) H_2_O_2_ intracellular expression; (**C**) c-MET inhibition; and (**D**) MCL-1 inhibition after treatments. Data are expressed as mean and error bars represent ± standard deviation; significance at *p* < 0.05 compared to negative control (HepG2 cells) and positive control (KCC-NH_2_-FA nanoparticles). In A and B, ^*^ is least significant and ^**^ indicates high significance. In C and D, ^*^ is least significant, ^**^ indicates medium significance and ^***^ a very significant effect compared to KCC-NH_2_-FA nanoparticles as control sample.

Caspase-3 activity is an important assay to evaluate the apoptosis induction pathway. In this study, we aimed to determine whether caspase-3 activity was enhanced when using a KCC-NH_2_-FA-CR–based nanoformulation for HepG2. As illustrated in Figure 10A, KCC-NH_2_-FA-CR treated HepG2 cells displayed a significantly stronger (p ˂ 0.05) caspase-3 activation than free CR and KCC-NH_2_-FA (no drug loaded carrier as control sample). The relatively high expression was 628.2 ± 5.6 pg/ml for KCC-FA-CR, 447.8 ± 6.0 pg/ml for free CR, 67.4 ± 9.2 pg/ml for KCC-NH_2_-FA, and 27.3 ± 15.3 pg/ml. Therefore KCC-FA-CR nanoformulation displayed a 1.4-fold enhancement of caspase-3 activity compared to free CR.

### Overproduction of intracellular H_2_O_2_ in HepG2 cells induced by CR nanoformulation

As shown in Figure 10B, the HepG2 cells treated with pure CR, KCC-NH_2_-FA MSNs, and KCC-NH_2_-FA-CR nanoformulations showed a significant change in H_2_O_2_ level (*p* < 0.05) compared to control HepG2 cells. Surprisingly, the KCC-NH_2_-FA-CR significantly increased the intracellular H_2_O_2_ production in HepG2 cells compared to pure CR, KCC-NH_2_-FA MSNs, and control HepG2 cells. The KCC-NH_2_-FA-CR nanoformulation showed good antioxidant activity when tested by 1-diphenyl-2-picrylhydrazyl free radical (DPPH•), and 2, 2-azino-bis (3-ethyl-benzothiazoline-6-sulfonic acid) diammonium salt (ABTS•) assays based on release experiments.

### Inhibition of c-MET in HepG2 cells induced by CR nanoformulation

HGF is a ligand for the receptor tyrosine kinase c-MET, with high affinity on liver cancer cells, and therefore activates numerous cellular signaling pathways in cancer development (e.g., signaling pathways in the proliferation process, motility, migration, invasion, mutation, protein overexpression, and others) [45]. Therefore, studying c-MET expression represents a promising approach for targeted cancer therapy. To assess inhibition of c-MET, we treated HepG2 cells with KCC-NH_2_-FA, CR, and KCC-NH_2_-FA-CR, and the results are shown in Figure 10C. Significant inhibition (*p* < 0.05) was obtained when the cells were treated with pure CR and KCC-NH_2_-FA-CR. Maximum inhibition was recorded when HepG2 cells were treated with 50 µg/ml of KCC-NH_2_-FA-CR (74.9 ± 0.7%) compared to pure CR at the same concentration (60.7 ± 0.8%), and a negligible effect with KCC-NH_2_-FA MSNs. Inhibition of c-MET, which has an anti-apoptotic effect, induces apoptosis as an anticancer mechanism. The present results indicate that KCC-NH_2_-FA-CR may induce apoptosis in HepG2 cells via this inhibition.

### Inhibition of MCL-1 in HepG2 cells induced by the CR nanoformulation

Targeting of the anti-apoptotic Bcl-2 family proteins is important for cancer treatment and preventing drug resistance. Among these proteins, MCL-1 is of particular interest [46]. We studied inhibition of MCL-1 by HepG2 cells treated with KCC-NH_2_-FA MSNs, CR, and KCC-NH_2_-FA-CR. The results (Figure 10D) showed that both pure CR and KCC-FA-CR significantly (*p* < 0.05) reduced MCL-1 in treated HepG2 cells compared to KCC-NH_2_-FA MSNs. When comparing the inhibitory effects of pure CR and KCC-NH_2_-FA-CR at a concentration of 50 µg/ml, we showed that the latter enhanced inhibition of MCL-1 to about 78%, whereas pure CR inhibited MCL-1 to about 67% only. The IC50 of MCL-1 value was ˃1000 μg/ml for KCC-NH_2_-FA MSNs without CR, 23.1 ± 2.6 μg/ml for pure CR, and 9.7 ± 0.1 µg/ml for KCC-NH_2_-FA-CR nanoformulations, respectively (Table 2). In addition, the KCC-NH_2_-FA MSNs showed negligible ability to suppress MCL-1 in HepG2 cells. The data from this assay showed that higher concentration led to higher suppression of MCL-1. These results also indicate that the KCC-NH_2_-FA-CR nanoformulation seems to be most promising as a MCL-1 inhibitor.

**Table 2:** IC50 concentration (µg/ml) of different treatments to inhibit c-MET and MCL-1 proteins in human HCC (HepG2) cells

	IC50 (µg/ml)	
Sample code	Molecular targets	
	**c-MET**	**MCL-1**
**KCC-NH2-FA**	>1000 ± 0.0	>1000 ± 0.0
**KCC-NH2-FA-CR**	8.4 ± 1.0	9.80 ± 0.04
**CR pure**	27.0 ± 3.7	23.15 ± 3.75

### Antioxidant activity of FA-conjugated nanoparticles loaded with prodrugs

The drug-loaded nanoparticles were evaluated for their antioxidant activities compared to pure form of tested prodrugs by means of two assays: ABTS.+ or DPPH.

### DPPH^.^ assay

As presented in Table 3, the antioxidant activity depended on type of prodrug (CR, QR or COL), type of MSN (KCC or MCC), and timing of sampling after the release experiment (1, 24, 48, 72, and 80 h). We detected no significant difference between pure CR and MCM-NH_2_-FA-CR for all time points. Comparing free CR with KCC-NH_2_-FA-CR at 1 h, 48 h, and 72 h, a significant difference (*p* ˂ 0.05) was observed, and the highest activity was 55 ± 1.7% at 72 h for KCC-NH_2_-FA-CR. In case of QR, there was a significant difference between QR loaded in both types (MCM and KCC) of MSN and QR only (*p* ˂ 0.05). The highest antioxidant activity was 72% after 24 h for KCC-NH_2_-FA-QR. In case of COL, only two samples showed significantly enhanced antioxidant activity: 51.2 ± 4.7% in MCM-FA-COL and 44.6 ± 3% in KCC-FA-COL. Finally, regarding drug type, the least antioxidant activity was found for COL, with CR yielding intermediate activity, and QR proving most effective. For both types of MSN, the antioxidant activity was most enhanced for KCC-NH_2_-FA loaded with CR, while both types were nearly equal for loaded QR and COL. These results demonstrate that the antioxidant activity as tested by DPPH depended on the type of MSN, type of prodrug, and timing.

**Table 3:** Antioxidant activity of pure and prodrug-loaded MSNs after drug release experiments in PBS at 37° C

Time (h)	DPPH			ABTS		
	Pure form	Drug-loaded MCM-FA-MSNs	Drug-loaded KCC-FA- MSNs	Pure form	Drug-loaded MCM-FA- MSNs	Drug-loaded KCC-FA- MSNs
**CR**						
	**CR**	**M-FA-CR**	**K-FA-CR**	**CR**	**M-FA-CR**	**K-FA-CR**
1	36.4 ± 5.3^bc^	39.9 ± 0.3^c^	53.0 ± 0.8^a^	46.7 ± 5.7^g^	77.5 ± 0.1^d^	83.2 ± 2.4^c^
24	32.5 ±1.0^c^	36.3 ± 3.0^c^	42.3 ± 4.2^c^	46.3 ± 7.9^g^	98.1 ± 0.8^a^	87.2 ± 2.6^b^
48	31.6 ± 3.2^c^	38.4 ± 2.3^c^	49.6 ± 0.2^b^	57.6 ± 0.2^f^	65.1 ± 1.7^e^	96.6 ± 0.2^a^
72	32.4 ± 3.3^c^	35.1 ± 3.5^c^	55.0 ± 1.7^a^	59.2 ± 0.9^f^	98.2 ± 0.1^a^	98.0 ± 0.0^a^
100	34.4 ± 2.3^c^	40.2 ± 4.1^c^	43.3 ± 4.4^c^	48.6 ± 2.4^g^	98.2 ± 0.3^a^	98.5 ± 0.1^a^
**QR**						
	**QR**	**M-FA-QR**	**K-FA-QR**	**QR**	**M-FA-QR**	**K-FA-QR**
1	33.6 ± 6.7^ce^	40.1 ± 3.1^ce^	40.6 ± 1.6^de^	35.4 ± 2.0^g^	63.3 ± 0.3^e^	89.7 ± 4.0^c^
24	28.5 ± 4.8^de^	44.8 ± 4.2^ce^	71.8 ± 0.7^a^	38.7 ± 2.5^g^	93.6 ± 2.9^bc^	95.6 ± 0.2^ab^
48	43.7 ± 1.1^ce^	52.3 ± 5.5^bc^	43.7 ± 0.8^ce^	55.1 ± 1.2^f^	94.1 ± 1.1^bc^	97.8 ± 0.3^ab^
72	37.5 ± 1.4^de^	38.3 ± 6.7^de^	35.9 ± 3.7^e^	65.3 ± 3.9^e^	98.1 ± 0.3^ab^	99.0 ± 0.2^a^
100	38.9 ± 3.4^de^	60.1 ± 4.6^b^	45.1 ± 3.4^cd^	50.6 ± 2.4^f^	84.7 ± 0.8^d^	95.1 ± 0.4^b^
**COL**						
	**COL**	**M-FA-COL**	**K-FA-COL**	**COL**	**M-FA-COL**	**K-FA-COL**
1	36.8 ± 4.6^abc^	31.5 ± 1.7^c^	31.5 ± 2.3^c^	6.3 ± 1.7^hi^	56.4 ± 5.0^bc^	60.0 ± 3.1^b^
24	28.0 ± 3.7^c^	31.6 ± 1.1^c^	29.8 ± 1.1^c^	11.1 ± 1.5^hi^	77.5 ± 6.0^a^	31.7 ± 1.4^g^
48	25.1 ± 2.0^c^	29.6 ± 0.0^c^	30.9 ± 0.2^c^	7.2 ± 1.6^hi^	57.6 ± 4.5^bc^	69.6 ± 2.9^a^
72	29.0 ± 0.5^c^	30.8 ± 0.2^c^	29.4 ± 0.9^c^	5.7 ± 0.7^i^	49.5 ± 4.1^cd^	45.1 ± 0.6^de^
100	26.8 ± 1.3^c^	51.1 ± 4.7^a^	44.6 ± 3.0^b^	18.0 ±1.9^h^	37.4 ± 2.1^fg^	38.7 ± 1.2^ef^

Data are expressed as mean ± standard deviation. The superscripts (i.e. ^a,b,c^) indicate whether the post hoc Fisher least significant difference (LSD) test (*p* < 0.05) shows a difference between mean values. Mean values sharing the same superscripts are not significantly different. Mean values with different superscripts are significantly different. Comparison is made both in rows, and in columns for each of the three pro-drugs. When two superscripts are presented, one of them can be used for comparison.

### ABTS.+ assay

The potential of CR, QR, and COL for scavenging ABTS.+ radical cations in their free form, drug loaded MSNs, and in FA-conjugated MSNs was evaluated. The results (Table 3) indicated that the nanoformulations based on both types of MSNs showed significant inhibition of ABTS.+ radical (*p* ˂ 0.05). Application of prodrug-loaded FA-conjugated MSNs increased the antioxidant activity compared to the respective pure forms. For CR, the highest antioxidant of activity 98.5 ± 0.1% was recorded for KCC-NH_2_-FA-CR after 100 h of release test. For QR, the highest activity was 99.0 ± 0.2% for KCC-FA-QR at 80 h of release. For COL, the loaded materials exhibited clear and significant antioxidant activity against ABTS.+ radical as well when compared to pure COL (*p* ˂ 0.05). These results are in line with those obtained previously, demonstrating that the prodrug-loaded nanoparticles are more effective in scavenging radical species compared to their pure forms [47–49]. QR and CR loaded into KCC-NH_2_-FA had the highest antioxidant activity, with negligible differences between the two, especially with longer times. In contrast to the DPPH assay, the ABTS assay showed that loading of COL to MSNs improved its antioxidant activity.

## DISCUSSION

In this paper, we test nanotechnology based targeted delivery of three natural anticancer prodrugs - CR, COL, and QR to fight HCC. Despite its being one of the leading cancers worldwide [22], most current strategies for treatment (e.g., combination therapy, radiation) are unsatisfactory [29]. Development of targeted drug delivery is widely regarded as a strategy to increase the efficiency of anticancer therapy and reduce negative side effects connected with chemotherapy. In this regard, nanotechnology offers the opportunity to develop drug carriers that could transport the needed drugs directly to and release them inside the cancer cells, so that overall drug doses are reduced and at the same time therapeutic efficiency is increased.

The results of the present study show that FA-conjugated MSNs with natural prodrugs loaded into the nanosized pores in these spheres are potentially highly effective nano-systems for targeted drug delivery and efficient at fighting HCC, and especially HepG2 cells. Below, we discuss in detail the properties of the anticancer MSNs from the perspective of their microstructure and the mechanism of their anticancer activity.

### Microstructure, physicochemical properties, and drug-loading capacity of MSNs for targeted drug delivery

Two types of silica nanoparticles were studied (Figure 1), KCC-1 (with diameter 197 ± 17 nm) and MCM-41 (with diameter 324 ± 33 nm) (Supplementary Table 1). Both types are characterized by a silica framework, and nanometer size pores with sizes in the range of 2 nm for MCM-Calcined and 3.4 nm for KCC-Calcined, according to our previous reports [42, 43]. A high reduction in the specific surface area was observed when prodrugs were loaded into the MSNs, which is an indication of the nanopores being filled by the prodrugs, in agreement with previous studies [14, 50–52].

The prodrugs loaded into the pores of MSNs took an amorphous form, as seen from DSC and XRD results (Figures 2 and 3). This is expected as embedding pro-drugs into pores of amorphous silica should prevent their crystallization during annealing. However, a small amount of pro-drugs close to the MSNs surface could crystalize, contributing to the weak XRD peaks for loaded MSNs.

This observation is in line with previous data obtained by [53, 54] for MSNs loaded with other drugs. For CR and QR, the FT-IR spectra showed some sharp peaks of low intensity corresponding to their pure spectra (Figure 4), which may indicate that a small fraction of these prodrugs is attached to the surface of nanoparticles in the crystalline form.

The drug-loading capacity depends on silica particles and drug type, as shown in Supplementary Figure 1 and Supplementary Table 1. For CR and QR, the drug-loading capacity was in the range 20–30 wt%. MCM-NH_2_-FA showed better loading capacity compared to KCC-NH_2_-FA, possibly because the MCM MSNs have a larger surface area and pore volume. The lowest loading content was found for COL, with 2.8% wt in MCM-NH_2_-FA-COL and 2.3% wt in KCC-NH_2_-FA-COL. One possible reason for the much smaller loading capacity of COL compared to the other prodrugs is its chemical structure, which could have led to a weak interaction with FA-conjugated and amino-modified MSNs.

The present results show the crucial role of combined amino functionalization and FA conjugation for successful cell targeting. Successful conjugation of FA was confirmed by FT-IR spectra, where new bands were observed from 1440 cm^-1^ to 1315 cm^-1^ (Figure 4). These bands might be the result of proton transfer from the carboxylic acid groups to amine groups on the surface of the modified MSNs [55, 56], and the amino groups functioned as bridges linking FA to the MSNs. Thus, we were able to construct a targeted nano-delivery system, where a considerable amount of prodrugs was enclosed in the nanopores of the MSNs, which protected the prodrug from interaction with the biological fluid environment and slowed its release into the cells.

For efficient cancer targeting, the MSNs need to be attracted by the cancer cells, which have a negative charge. A high positive zeta potential of FA-conjugated MSNs is advantageous for targeting cancer cells because it leads to electrostatic attraction to the the negatively charged cancer cell membrane. The charge of MSNs determines nanoparticle uptake by cells and escape from endosomal entrapment [57]. It also affects loading of drugs and sustained release from their carriers [58]. The as-synthesized (calcined) MSNs display a high negative zeta potential of -40 mV at a physiological pH 7. The amine-modified MSNs showed a positive zeta potential ranging between +30 to +50, which is mainly because of amino groups on the MSN surface. The FA conjugation led to a further increase of the zeta potential to about +40, as shown in Figure 5. This increase can be explained by FA attachment to amino groups, which contribute to a high positive zeta potential. As a consequence, receptor-mediated endocytosis internalization of MSNs in cells takes place, as shown in Figures 7 and 8. Our results are in accordance with previous data for other drugs loaded to FA-conjugated MSNs [59, 60].

### Cellular uptake of MSNs by HeLa cells

Once the drug-loaded MSNs become attracted to the cancer cell membranes, the key success factor for their anticancer activity is cellular uptake of the drug nanocarriers (or in other terms nanocarrier internalization) [61]. Many factors (i.e., surface of nanoparticles, timing, size) may influence cellular uptake of nanoparticles by cancer cells. We studied three factors: type of MSNs (MMS vs KCC), kind of surface modification (as-synthesized MSNs, amino-modified MSNs, and FA-conjugated MSNs), and incubation time (4, 12, 24, and 48 h). More efficient cellular uptake was observed for FA-conjugated MSNs compared to non-modified and amino-modified versions, as shown in Figure 6. As SEM observation showed, the cell’s membranes were still intact and distinct (Figure 6) after MSN internalization. Our results are in agreement with previously published studies for cancer targeting using FA ligands with other MSNs [20, 21, 61]. Such an efficient uptake can be explained by the folate-receptor–mediated endocytosis effect [62, 63], which can be simply explained by interaction of the FA ligands conjugated on MSNs with the folate receptors expressed on membranes of HepG2 cancer cells. A mechanism by which MSNs, especially KCC MSNs, could become entrapped in cells was proposed by [64] and [65], and consists of the following steps: the MSNs are trapped by intracellular organelles and subsequently escape from endosomes/lysosomes into cytoplasm and nucleus.

An important finding is that the two silica nanoparticles used in our studies behaved differently in terms of dispersion and aggregation when they were internalized by the cells. From the SEM and CLSM observations, the MSNs of the two types were mainly accumulated in the perinuclear region of cells and cytoplasm, in agreement with other studies by [66, 67]. Only few of them were observed inside the nucleus (blue) (Figure 7). The MCM-based nanoparticles (Figure 6A, 6C, 6E) were seen in large vesicles as aggregated clusters, while the KCC-based nanoparticles (Figure 6B, 6D, 6F) were distributed uniformly in the cells without aggregation. The reason for such a difference in MSN behavior was the stronger aggregation of MCM compared to KCC. As seen in Figure 1, the KCC MSNs were dispersed as single particles, while KCC. MSNs showed a tendency to aggregate. Relatively large aggregates of MCMs may display smaller mobility within the cells than dispersed KCC MSNs. These results indicate that the fine details of the MSN structure and surface chemistry may influence cellular uptake and should be taken into account for selection of silica type for designing nano-delivery systems in medical applications.

As far as the incubation time effect is concerned, CLSM studies (Figure 7) showed that a longer time of incubation (48 h) led to a higher accumulation and internalization of nanoparticles into cancer cells. This observation may indicate that the uptake rate-controlling factor was diffusion of the MSNs towards the cells. The electrostatic interactions would attract FA-conjugated MSNs towards cancer cells, which would become internalized and penetrate the cells in an increasing amount with increasing exposure time.

Our results show that the KCC-MSNs are more favorable compared to MCM-MSNs as an efficient drug delivery system in cancer therapy: they exhibited better uptake, distribution, and higher penetration into the cells. Even though KCC MSNs were about twice the size of the MCM MSNs, due to their less aggregation tendency, they were more mobile. For the same reason, the KCC-based MSNs probably showed better uptake than MCM-based MSNs.

### Intracellular release of prodrugs form MSNs in HeLa cells

Following cellular internalization of nanoparticles inside tumor cells, another key factor for drug efficiency is whether the drug loaded into MSNs can be efficiently released [68]. For CLSM studies, we chose CR because it exhibits inherent green fluorescence, and direct observation is possible for cellular uptake and release behavior (Figure 8). CR is a small molecule, so it diffuses rapidly across all cell organelle membranes. After a few hours, most of these molecules were distributed in all cell compartments, while after a longer time (24 and 48 h), they accumulated inside the nucleus. Such a diffusion process for small free anticancer drugs was proposed by [64] for doxorubicin. Of interest is the path of the MSNs after they become internalized into cancer cells. CR loaded in both types of MSNs (MCM-NH_2_-CR, MCM-NH_2_-FA-CR, KCC-NH_2_-CR, and KCC-NH_2_-FA-CR) was found in the cytoplasm and some in the nucleus, as indicated by the green dots in Figure 8. MCM-NH_2_-CR and MCM-NH_2_-FA-CR showed intense green fluorescence, which indicates that, a large fraction of CR still within the MSNs pores, and that the release of CR from both MCM samples requires a long time. Thus, we infer that with release of CR, a distributed green fluorescence intensity should be observed. The KCC-NH_2_-FA-CR showed a slow release of CR over time, as indicated by red arrows (lighter/distributed green fluorescence) compared to MCM-NH_2_-FA-CR (darker/non-distributed green fluorescence) in Figure 8. Where the distributed green fluorescence may correspond to CR released from particles into the cells.

The slow release of drug loaded into MSNs would be expected because CR molecules are embedded in MSN nanopores and can leave the pores only gradually. The result is a steady delivery of the drug over an extended period of time compared to application of free prodrug, where the initial concentration is high, but it rapidly decays. Such an effect is beneficial for killing cancer cells because the drug is delivered to critical sensitive sites in cancer cells in a constant manner.

Differences in intracellular pH in cell organelles may also be a factor influencing CR release; an acidic medium might offer protons bridging the link between drugs and amine-modified nanoparticles, disrupting their connection and facilitating release of drugs. The slower release for MCM MSNs comparing to KCC MSNs may result from smaller pore size of the MCM MSNs comparing to KCC MSNs. Further, KCC MSNs were uniformly distributed and more uptake within the cancer cells, comparing to MCM ones. Hence we think that drug release takes place also in crucial cells organelles, like nucleus, which may contribute to their higher efficacy comparing to MCM ones. CR-loaded MSNs thus may ensure sustained intracellular release, leading to prolonged therapeutic anticancer activities, as in another recently studied anticancer drug [69]. It could be expected that the drug loaded to both types of MSNs – QR and COL – possess similar uptake and intracellular release mechanisms to CR-loading because they were prepared in a similar manner and have a similar nanostructure.

### Anticancer activity evaluations

The anticancer activity of the MSNs correlated well with the results of pro-drug release studies. The cytotoxicity tests clearly showed the advantage of FA-conjugated MSNs, both MCM-NH_2_-FA-CR and KCC-NH_2_-FA-CR, loaded with CR, comparing to amine-modified ones. The importance of FA conjugation to other types of MSNs for improving the anticancer effect for other drugs was reported by [61, 62, 67, 68, 70]. KCC-NH_2_-FA-CR displayed higher anticancer activity compared to MCM-NH_2_-FA-CR. The enhanced cytotoxicity was observed for both a HepG2 as well as for HeLa cancer cells (Figure 9).

The reason for the reduction in the applied drug doses when delivered via MSNs compared to the free form is probably the extended duration of cell exposure to the prodrugs, slowly released from the nano-channels in the MSNs.

### Apoptosis induction in human HCC cells

For further studies of the mechanism of action and the molecular signaling pathways leading to apoptosis of human HCC cells, we selected CR and its nanoformulation KCC-NH_2_-FA-CR, which showed the best performance in all tests listed above. Apoptosis as one of the main anticancer mechanisms relies on the caspase activation pathway as a primary route [71], and several secondary signaling pathways may contribute to apoptosis induction for killing cancer cells.

### Activation of caspase-3 in human HCC cells

Figure 10A demonstrates the potential of KCC-NH_2_-FA-CR to activate caspase-3 compared to pure CR and positive control sample KCC-NH_2_-FA MSNs. Our results indicate that the mechanism of action for KCC-NH_2_-FA-CR is in agreement with that proposed by [72, 73]. KCC-NH_2_-FA-CR can activate caspase-3 as the main route for apoptosis; therefore, KCC-NH_2_-FA-CR is a promising therapeutic nano-agent target for human HCC treatments with a selective anticancer mechanism. In the following sections, we discuss the possible secondary routes (signaling pathways) that may contribute to apoptosis through their roles in activation of caspase-3.

### H_2_O_2_ intracellular production in human HCC cells

There is evidence [74] for an important role of H_2_O_2_, which is considered an important signaling molecule in cancer development, with a dual function. H_2_O_2_ is produced in high amounts in cancer cells during several stages of cancer progression (e.g., DNA development, cell proliferation, anti-apoptosis, metastasis, hypoxia). Growing evidence also shows that H_2_O_2_ is an important molecule for inducing apoptosis in cancer cells, and several anticancer drugs in clinical use are based on H_2_O_2_ [74]. Recently, strategies to fight cancer by exploiting ROS, among them H_2_O_2_ [75], have been developed. Cancer cells show greater ROS accumulation compared to normal cells [76], including levels of H_2_O_2_. Discussions have focused on whether decreasing or increasing H_2_O_2_ is better for cancer treatment [75]. However, in recent years, the trend has been to increase H_2_O_2_ levels by applying anticancer drugs or an external source of H_2_O_2_ such as radiation therapy to target cancer cells.

The present results show that H_2_O_2_ level is increased in HepG2 cells after treatment with KCC-NH_2_-FA-CR compared to pure CR (Figure 10B). Thus, a further plausible reason for their anticancer effect is penetration of KCC-NH_2_-FA-CR into the cytoplasmic regions of cells. The released CR may accumulate directly in mitochondria and lead to increased H_2_O_2_ in these organelles via induction of enzymes regulating this function. This observation is in the line with other studies that showing the importance of selected anticancer drugs in increasing the intracellular expression of H_2_O_2_ for killing several cancer cells [77, 78]. The results from this study show a possible role of the nano-targeted system KCC-NH_2_-FA-CR for overproducing intracellular H_2_O_2_ in specific sites in cancer cells, compared to direct use for free prodrugs. Therefore, KCC-NH_2_-FA-CR offers a good opportunity for targeting the human liver cancer cell line (HepG2).

### c-MET inhibition in human HCC cells

In considering the mechanism of killing cancer cells, it is necessary to study alternative pathways of signal transduction in pathogenesis of HCC. Including the c-MET pathway is important because it is a known high-affinity ligand [79]. Therefore, a drug agent capable of selectively targeting c-MET is promising for liver cancer therapy. Much attention has focused on novel tyrosine kinase inhibitors, as well as drugs for c-MET inhibition such as sorafenib, approved by the FDA. CR in pure form can block HGF-induced signaling by specific inhibition of the c-Met/ERK/Snail pathway in prostate cancer cells [80]. However, these drugs have some limitations [29]. Our results confirm possible effect of CR on inhibition of c-MET in HepG2, and furthermore, stronger effect when CR is delivered using KCC vehicle (Figure 10C). The mechanism by which the nano-targeted MSN KCC-NH_2_-FA-CR inhibits c-MET could be reduction of cellular proliferation and induction of apoptosis via overproduction of p53, as proposed by [81] and [82]. Therefore, the obtained results for inhibiting c-MET by the KCC-NH_2_-FA-CR nano-system shows its ability to extend the selective killing, specific targeting and induce apoptosis in HCC compared to pure CR with non-specific targeting, and random cellular uptake.

### MCL-1 inhibition in human HCC cells

To provide additional information concerning the main pathway for apoptosis caused by the anticancer activity of the studied MSNs in HepG2 cells, we assessed one of the anti-apoptotic family proteins. Many anti-apoptotic proteins play an important role in hematological malignancies and in solid tumors because of their upregulation, among them MCL-1 protein, leading to resistance to apoptosis in cancers [83]. MCL-1 is a very short half-life protein in comparison to other Bcl-2 family members, making it a promising target for cancers [28, 46]. In cancer development, MCL-1 is upregulated, preventing apoptosis; thus, downregulation or knockdown of MCL-1 contributes to apoptosis, as reported in several cancers [84]. CR alone shows down regulation of MCL-1 in acute myelogenous leukemia [85]. We expected that our targeted system would down regulate more efficiently than when CR is used in the pure form because it releases the CR prodrug to different organelles in cells. The results from our study (Figure 10D) showed that the KCC-NH_2_-FA-CR nanoformulation causes an efficient down regulation of MCL-1 in HepG2 cells compared to free CR. This result confirms that the high anticancer activity of KCC-NH_2_-FA-CR may be also indirectly related to its ability to down regulate c-MET in HepG2 cells, where the MCL-1 is the key protein for tumor survival. Inhibition of MCL-1 also contributes indirectly to inducing apoptosis by supporting the activation of caspase-3, which can be correlated with activation of caspase-3 in HepG2 cells (Figure 10A). Future studies should consider targeted nano-systems over simple pure drug application alone.

In summary, the anticancer mechanism of the KCC-NH_2_-FA-CR nanoformulation leading to HepG2 apoptosis seems to be activation of caspase-3. Caspase-3 activation contributes to other signaling pathways (secondary routes), including overproduction of H_2_O_2_ and inhibition both of c-MET and MCL-1.

**Figure 11:**
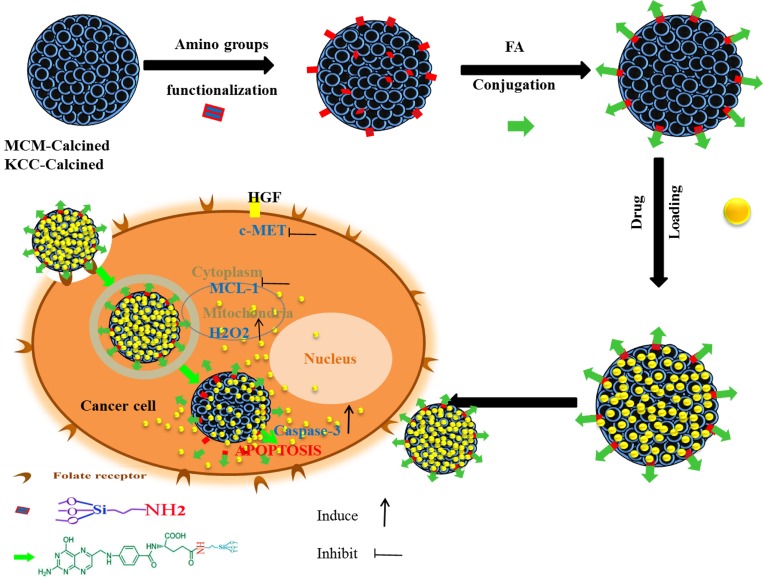
Schematic representation of the prepared nano-system from preparation, internalization, and anticancer mechanism of action in human liver carcinoma (HepG2) cells This schematic shows the prodrug release into cancer cells and the main anticancer action for inducing apoptosis via activation of caspase-3 for killing HepG2 cancer cells, proposed by assistance from important signaling pathways (c-MET, MCL-1, and H_2_O_2_).

Figure 11 shows in a schematic way the preparation of the MSNs based anticancer pro-drug carriers and the mechanism of their action. Amino groups and subsequent folic acid functionalization leads to attract of the MSNs towards cancer cells, and their internalization. Prodrugs loaded into the MSNs pores come out into the HepG2 cells, and reach cytoplasm, mitochondria, as well as nucleous. Cells apoptosis occurs, due to activate of caspase-3, increase of H_2_O_2_ level, and inhibition of MCL-1 and c-MET signaling pathways.

### Antioxidant activity evaluations

In addition to the anticancer activities and possible mechanisms of action, we provide evidence that the nano-delivery system with MSNs exhibits strong antioxidant activity when compared to free prodrug under the same conditions. In fact, antioxidants prevent cancer development in several ways, such as delay, prevention, and even removal of oxidative damage from ROS, as reported by [86]. Our results, as presented in Table 3, demonstrate that the antioxidant activity of prodrug-loaded nanoparticles is enhanced compared to pure forms of them. Enhancement of radical scavenging (of DPPH and ABTS.+ free radicals) depended on release time, material type, prodrug type, and even the kind of assay (DPPH and ABTS). In this context, QR and its nanoformulations) MCM-NH_2_-FA-QR and KCC-NH_2_-FA-QR) exhibited the strongest antioxidant activities, followed by CR nanoformulations (MCM-NH_2_-FA-CR and KCC-NH_2_-FA-CR) and COL nanoformulations (MCM-NH_2_-FA-COL and KCC-NH_2_-FA-COL). Therefore, QR-loaded MCM and KCC MSNs could be of great interest as strong natural antioxidants. The enhancement in antioxidant activity also shows that such nano-delivery systems may exert a strong anti-inflammatory action because the antioxidants are related to various biological roles in *in vitro* and *in vivo* studies.

In summary, we present a simple and effective targeted drug delivery system in which MSNs are used as vehicles to transport the natural prodrugs CR, QR, and COL into HepG2 cancer cells. Two types of MSNs were studied: KCC-1 and MCM -41, evaluated as-synthesized, amine-functionalized, or in a FA-conjugated state. The FA conjugation of the MSNs efficiently enhanced their internalization by HeLa cells. The KCC-type MSNs exhibited higher intracellular uptake than MCM-type MSNs. Also, KCC type MSNs were uniformly distributed in the cells, including in the nucleus and mitochondria. Comparing anticancer efficiency during *in vitro* tests of the FA-conjugated prodrug-loaded MSNs, the most efficient prodrug was CR (KCC, FA conjugated, IC50 level 15.6 μg/ml), followed by QR (KCC, FA conjugated, IC50 level 16.8 μg/ml), and then COL (MCM, FA conjugated, IC50 level 19.1 μg/ml). The mechanism of cell death is through apoptosis induction. Caspase-3 activation is proposed to contribute to other signaling pathways (secondary routes), including overproduction of H_2_O_2_ and inhibition both of c-MET and MCL-1. FA-conjugated MSNs loaded with prodrug, especially KCC MSNs, displayed a high free radical scavenging activity, with antioxidant activity ranked as follows: QR>CR>COL. Thus, the CR nanoformulation based on KCC type MSNs would have great potential for HCC treatments through the apoptosis induction pathway. By using MSNs as nanocarriers, it was possible to overcome the barrier of poor water solubility and delivery difficulties to target specific cancer cell organelle sites. This possibility opens perspective on the use of this inexpensive and safe prodrug in cancer therapy application.

## MATERIALS AND METHODS

### Materials

The detailed on all used materials are described in support information section.

### Synthesis and modification of MSNs

Two types of MSNs were studied, one denoted as MCM-41 (MCM) and the other as KCC-1 (KCC).

### Synthesis of MCM and MCM-FITC nanoparticles

MCM nanoparticles were synthesized according to a previously reported method [87] with minor modifications. The preparation procedure of the MCM-41 nanoparticles was described in our previous report [43]. To prepare green fluorescence–labeled nanoparticles, the co-condensation route was used as reported previously [87], and these nanoparticles are designated as MCM-FITC. The synthesis method is fully described in the Supplementary Information (SI).

### Synthesis of KCC and KCC-FITC nanoparticles

The KCC type material was as reported in [88] with some modifications as reported in [42]. To produce green fluorescence–labeled nanoparticles, the post-synthesis route was employed by modifying the procedure reported in [89]. Such MSNs are designated as KCC-FITC, and their production method is described in the Supplementary Information (SI).

### Functionalization of MSNs

The amine-functionalization was performed according to [90] with a slight modification as described in our previous reports. The difference is the amount of APTES used (2.5 ml instead of 1.5 ml). The obtained materials were designated as MCM-NH_2_ and KCC-NH_2_.

### Folic acid conjugation of MSNs

FA conjugation was performed for the amine-functionalized MSNs. A two-step procedure reported in [87] was used. FA-conjugated MSNs are designated as KCC-NH_2_-FA and MCM-NH_2_-FA.

### Drug loading into MSNs

For prodrug loading, we used the FA-conjugated MSNs with amine-functionalized MSNs for comparisons). In case of FA-conjugated MSNs (MCM-NH_2_-FA and KCC-NH_2_-FA), a modified procedure of the solvent evaporation method reported by [91] for drug loading was employed to load the three prodrugs (CR, QR, and COL). The resulting materials were designated as MCM-NH_2_-FA-CR, MCM-NH_2_-FA-QR, and MCM-NH_2_-FA-COL, and as KCC-NH_2_-FA-CR, KCC-NH_2_-FA-QR, and KCC-NH_2_-FA-COL.

In case of amine-functionalized MSNs (MCM-NH_2_ and KCC-NH_2_), a similar manner of preparation as described in our previous reports was used. The resulting materials were designated as MCM-NH_2_-CR, MCM-NH_2_-QR, MCM-NH_2_-COL, KCC-NH_2_-CR KCC-NH_2_-QR, and KCC- NH_2_-COL. Below we list the samples used in the present study.

As-synthesized MSNs: MCM-Calcined and KCC-Calcined.

Amine-modified MSNs: MCM-NH_2_ and KCC-NH_2_

FA-conjugated MSNs: MCM-NH_2_-FA and KCC-NH_2_-FA.

FA-conjugated MSNs with prodrugs loaded: for CR: MCM-NH_2_-FA-CR and KCC-NH_2_-FA-CR; for QR: MCM-NH_2_-FA-QR and KCC-NH_2_-FA-QR; and for COL: MCM-NH_2_-FA-COL and KCC-NH_2_-FA-COL.

Amine-modified MSNs with prodrugs loaded: for CR, MCM-NH_2_-CR and KCC- NH_2_-CR; for QR, MCM-NH_2_-QR and KCC-NH_2_-QR; and for COL: MCM-NH_2_-COL and KCC- NH_2_-COL.

Dye fluorescence–labeled MSNs: MCM-FITC and KCC-FITC.

### Physicochemical characterizations of the MSNs

Several techniques used during our investigations to characterize the MSNs are fully described in the Supplementary Information.

### Cell cultures

Two cancer cell lines, the HCC HepG2 line (VACSERA, Egypt) and human cervical cancer cell line HeLa (Sigma-Aldrich, ECACC), were used for *in vitro* biological evaluations. The cells were cultured in DMEM supplemented with 10% fetal bovine serum, penicillin G (100 U/ml), and streptomycin (100 µg/ml). The cell cultures were maintained at 37° C in a humidified 5% CO_2_ atmosphere.

### Cellular uptake study by means of SEM

The HeLa cells (5 × 10^5^ cell/well) were grown on sterile glass coverslips (diameter, 12 mm) to sub-confluence in 24-well cell culture plates and allowed to attach for a 24-h incubation period. Subsequently, the cells were treated with MCM-Calcined, MCM-NH_2_, KCC-Calcined, KCC-NH_2_, MCM-NH_2_-FA, and KCC-NH_2_-FA in DMEM at a concentration of 100 µg/ml in culture medium for 24 h. After that, the treated cells were washed with PBS and then fixed with 4% paraformaldehyde in PBS buffer at room temperature for 2 h; thereafter, the cells were dehydrated with graded ethanol (50, 60, 70, 80, 90, and 100%) for 30 min each and dried under a laminar chamber overnight. Finally, the coverslip cultures were sputter-coated with gold-palladium via a sputter coater (SCD 005, Bal-Tech), and cells were examined using a field emission electron microscope (Ultra Plus, Zeiss, Germany) operating at 2 kV.

### Cellular uptake and intracellular drug release studied by means of confocal laser scanning microscopy (CLSM)

The cellular uptake of green fluorescence–labeled MSNs and intracellular release of drug-loaded MSNs were visualized by confocal microscopy in addition to FITC dye. To perform the intracellular release studies, we used pure CR and loaded MSNs because CR has green autofluorescence properties compared to other prodrugs; so that this property enable us to direct visualization with CLSM like a dye molecules. Briefly, HeLa cells (5 × 10^5^ cell/well) were seeded onto sterile glass coverslips (diameter, 12 mm), mounted in 24-well cell culture plates, and incubated for 24 h to allow attachment. Subsequently, the MCM-FITC, KCC-FITC, CR, MCM-NH_2_-CR, KCC- NH_2_-CR, MCM-NH_2_-FA-CR, and KCC-NH_2_-FA-CR suspensions in culture medium at a final concentration of 15 µg/ml were added. After incubation for 4 h, 12 h, 24 h, and 48 h, the medium was removed, and the cells were washed with PBS (pH 7.4) three times. Afterwards, the cells were fixed with 4% paraformaldehyde for 15 min at room temperature, washed with PBS and then deionized water, and dried and mounted onto the glass slides with mounting medium containing DAPI for nuclei staining. The samples were dried in the dark for 24 h before confocal imaging. Finally, samples were analyzed using confocal laser scanning microscopy (Carl Zeiss Microscopy, GmbH, Jena, Germany) with 40/100× water objective at 488 nm excitation and 500–550 nm emission for detecting the FITC green fluorescence dye and the green fluorescence of the anticancer CR compound channel, and 405 nm excitation with 420–840 nm emission for detecting the DAPI channel. After being imaged, the samples were stored at −20° C.

### *In vitro* cytotoxicity evaluations

Anticancer efficiency and cyto-biocompatibility properties were assessed based on the MTT assay [92] against HepG2 cells. The HepG2 cells were seeded in 96-well tissue culture plates at a density of 2 × 10^5^ cells/ml per well. After a 24-h period of incubation at 37° C in a humidified 5% CO_2_ atmosphere, cell monolayers were confluent; the medium was removed from each well and washed and replaced with fresh DMEM medium containing 100 µl PBS and various concentrations (1, 5, 50, and 150 µg/ml) of free CR, QR, and COL, as well as prodrug-loaded to FA-conjugated MSNs (MCM-NH_2_-FA-CR, KCC-NH_2_-FA-CR, MCM-NH_2_-FA-QR, KCC-NH_2_-FA-QR, MCM-NH_2_-FA-COL, and KCC-NH_2_-FA-COL) and amino-modified MSNs (MCM-NH_2_-CR, KCC-NH_2_-CR, MCM-NH_2_-QR, KCC-NH_2_-QR, MCM-NH_2_-COL, and KCC-NH_2_-COL) for comparison. During the biocompatibility evaluation for as-synthesized (MCM-Calcined, KCC-Calcined) and amine-modified MSNs (MCM-NH_2_ and KCC-NH_2_), concentrations of 25, 50, 250, and 750 µg/ml were used. Additionally, for cell controls, 100 µl of medium was used. All cultures were incubated for 24 h in the same conditions. Cell morphology was observed microscopically (inverted light microscopy) for detectable morphological alterations, such as loss of confluency, cell rounding and shrinking, and cytoplasm granulation and vacuolization. Thereafter, the medium was removed from all wells, fresh DMEM medium containing 50 µl of MTT (1 mg/ml) solution was added to each well, and the plates were incubated for 4 h at 37° C. After discarding the medium containing MTT solution, 100 µl of DMSO was added to each well to dissolve MTT formazan crystals. Afterwards, the plates were gently shaken for 5 min to ensure that the crystals were completely dissolved. Finally, the absorbance of formazan product was read by an ELISA reader at 540 nm.

### Caspase-3 activity assay

The caspase-3 activity for free CR, KCC-FA, and KCC-FA-CR was measured and compared to controls (HepG2 cells without any treatment) using the human active caspase-3 content assay kit (Invitrogen Ca3; Active), according to the manufacturer’s protocol. Cells were cultured onto 96-well plates to density (1.2–1.8 × 10,000 cell/well) in a volume 100 µl of complete growth medium (RPMI 1640 containing 10% fetal bovine serum at 37° C) and treated with 100 µl of the tested compound per well for 24 h before the enzyme assay. The absorbance was recorded at 450 nm on a Robonik P2000 ELISA reader. The assay was done in triplicate, and the data are expressed as mean ± standard deviation.

### H_2_O_2_ assay

The H_**2**_O_2_ assay kit from Abcam (ab102500) was used according to the manufacturer’s instructions (Abcam, UK) to study intracellular expression in HepG2 cells treated with 100 μl of each sample and incubated for 24 h. In brief, after treatment and incubation of cells with CR, the KCC-NH_2_-FA and KCC-NH_2_-FA-CR MSNs were harvested, homogenized, and centrifuged, and the clear supernatant was used for the colorimetric assay. The reaction reagent for the assay (50 μg/ml) was mixed into each well and incubated at room temperature for 10 min, protected from light. Finally, the absorbance was measured by a microplate reader at 570 nm (Robonik P2000 ELISA reader). Each tested sample was done in triplicate. The mean optical density values were plotted on the standard curve, and the results were estimated.

### ELISA c-MET assay

The c-MET assay in our study was done based on the ELISA kit according to the manufacturer’s instructions in the standard protocol from Abcam (ab126451). After treatment and incubation with CR, the KCC-FA and KCC-FA-CR cells were harvested, lysed, and centrifuged for 10 min at 2–8° C (13,000 rpm). The supernatant was separated and assayed for c-MET with standard curve using 100 μl of the supernatants and standards. After incubation for 1 h at room temperature and three washes, 100 μl of anti-Met (Tyr1234/1235) was added. The samples then were incubated for 1 h at room temperature with shaking and washed three times, and then 100 μl of prepared 1× horseradish peroxidase–conjugated anti-rabbit immunoglobulin G was added to each well. Following incubation for 1 h at room temperature with shaking, samples were washed, and 100 μl of 3,3′,5,5′-tetramethylbenzidine (TMB) substrate solution was added. After a further incubation for 30 min in the dark with shaking, 50 µl of stop solution was added to each well, and the absorbance at 450 nm was measured immediately. All samples were analyzed in triplicate.

### ELISA MCL-1 assay

The MCL-1 assay was performed using an ELISA kit (Cell Signaling Technology, USA), following the manufacturer’s instructions. Samples and standards (100 μl) were added into appropriate wells and incubated for 2.5 h at room temperature with shaking. After that, wells were washed four times, and then 100 μl of prepared 1× detection anti-MCL1 antibody was added, followed by incubation again for 1 h at room temperature. Subsequently, the wells were washed again, and 100 μl of prepared 1× horseradish peroxidase–conjugated anti-rabbit immunoglobulin G was added to each well. Wells were incubated for a further 1 h at room temperature and washed, and 100 μl of TMB One-Step Substrate Reagent (Item H) was added to each well. After incubation for a further 30 min at room temperature in the dark, the stop solution (50 μl) was added, and this mixture was measured at 450 nm immediately. All samples were analyzed in triplicate.

### Antioxidant acx`tivity measurements

We performed the antioxidant properties based on release experiments. They were intended to select a high antioxidant by means of time intervals. Different *in vitro* release experiments were carried out in PBS medium at 37° C, pH 7. The pure CR, QR, and COL and prodrugs-loaded FA-conjugated MSNs (MCM-NH_2_-FA-CR, KCC-NH_2_-FA-CR, MCM-NH_2_-FA-QR, KCC-NH_2_-FA-QR, MCM-NH_2_-FA-COL, and KCC-NH_2_-FA-COL) were exposed to PBS solution for different intervals (1, 24, 48, 72, and 100 h). Subsequently, samples were centrifuged at 10,000 rpm to precipitate the MSNs used as carrier. Finally, all obtained samples were used for antioxidant determination with two standard assays.

### DPPH· scavenging activity

The DPPH· scavenging potential of all investigated samples was determined using a modified method according to [48, 93] with minor modifications. Briefly, 1 ml of DPPH· (100 μM) dissolved in ethanol was added to equivalent aliquots of samples and subsequently vortexed and left to incubate at room temperature in the dark for 30 min. Absorbance was recorded at 517 nm with a UV-Vis spectrophotometer (Cary 300, Agilent Technologies). For control samples, the same amount of PBS buffer was used, and a similar approach was followed as described above with the real samples. The free radical scavenging activity was calculated using Eq. (1):

DPPH⋅ Scavenging%=(Abs. of control sample−Abs. of tested sample/Abs. of control sample)×100.

### ABTS.+ scavenging activity assay

The ABTS.+ scavenging capacity was measured according to a method described previously [49, 94]. First, the ABTS.+ stock solution was prepared in PBS buffer (7 mM ABTS). To prepare the ABTS.+ radical cation solution, the ABTS stock solution was mixed with 4.9 mM potassium persulfate solution in equal quantities; subsequently, the mixture solution was allowed to stand at room temperature in the dark for 16 h. Next, the prepared ABTS.+solution was diluted with PBS (pH 7.4) to an absorbance of 0.70 (± 0.02) at 734 nm. An equivalent quantity of all pure and loaded material samples (described above) was reacted with 1 ml of the ABTS.+ solution at 30° C in the dark for 5 min. The absorbance was then measured at 734 nm using a UV-Vis spectrophotometer. PBS was used in the control sample instead of the real sample to be used in the ABTS.+ scavenging percentage from Eq. (2):

ABTS.+Scavenging%=(Abs. of control sample−Abs. of tested sample/Abs. of control sample)×100.

### Statistical analysis

Statistical analyses were done using SPSS software (SPSS 16.0 for Windows; SPSS, Inc., Chicago, IL, USA); for data shown in Figure 9, each test was for samples done in triplicate. One-way ANOVA with post hoc Fisher’s least significant difference test (*p* < 0.05) was performed for the data shown in Figure 10 and Table 3, for samples analyzed in duplicate and according to web-based software [95].

## SUPPLEMENTARY MATERIALS FIGURES AND TABLE


