# Supplementary material for: Folic acid–conjugated mesoporous silica particles as nanocarriers of natural prodrugs for cancer targeting and antioxidant action
Source: Oncotarget. 2018 May 29;9(41):26466–90. doi: 10.18632/oncotarget.25470 (PMC5995188; doi:10.18632/oncotarget.25470)
Supplement: Supplementary file 1 [file oncotarget-09-26466-s001.pdf]

Folic acid–conjugated mesoporous silica particles as nanocarriers of natural prodrugs for cancer targeting and antioxidant action

SUPPLEMENTARY MATERIALS

Materials

Cetyltrimethylammonium bromide was obtained from Fluka; tetraethyl orthosilicate (98%), QR, CR, COL, sodium hydroxide, cetylpyridinium bromide (CPB), cyclohexane, isopropanol, urea, 3-aminopropyltriethoxysilane (APTES; 99%), paraformaldehyde, FA (98%), 1, 1-diphenyl-2-picrylhydrazyl free radical (DPPH·), and 2, 2-azino-bis (3-ethyl-benzothiazoline-6-sulfonic acid) diammonium salt (ABTS, >98%) were purchased from Sigma-Aldrich; acetone and methanol were from Alpha Chemica (India); ethanol from Sham lab (Syria); toluene anhydrous from POCH (Poland); dimethyl sulfoxide (DMSO)/HPLC grade from Tedia (USA); fluorescein isothiocyanate (FITC), N-hydroxysuccinimide, and 1-(3-dimethylaminopropyl)-3-ethylcarbodiimide hydrochloride from Across Organics; and phosphate-buffered saline (PBS), Dulbecco's modified Eagle's medium (DMEM), fetal bovine serum, penicillin G, streptomycin, and 4′,6-diamidino-2-phenylindole (DAPI) were purchased from Life Technologies, Poland. Ultrapure water (18.2 MΩ, Millipore) was used in the preparation of all aqueous solutions and experiments. All analytical and reagent grade materials were used as purchased.

MCM-FITC MSN

Firstly, FITC (12.5 mg) was dissolved in absolute ethanol (7 ml), then APTES (100 µL) was added under stirring and the mixture was stirred for 2 h. After that, TEOS (5 mL) was added slowly dropwise to the mixture solution and stirred for another 20 min to obtain (TEOS/FITC/APTES). In another container, CTAB (1 g) was dissolved in a ultrapure water (480 mL) and sodium hydroxide (2 M, 3.5 mL) then was added and heated up 80° C and stirred vigorously for 2 h (CTAB solution). Subsequently, the TEOS/FITC/APTES solution was slowly added to the mixture of CTAB solution and stirred for 2 hours later under the same conditions, then the solution was cooled down to room temperature and the particles were isolated by centrifugation and washed several times with methanol until all the unbounded FITC was removed. The silica particles were allowed to dry at room temperature overnight. The surfactant was removed from the pores of the silica particles by alcoholic-acidic extraction, as-synthesized silica particles (1 g) were dissolved in a solution of methanol (100 mL) and hydrochloric acid (12.1 M, 6 mL) and refluxed for 24 hours. The particles were then filtered and washed thoroughly to remove the surfactants and unbound FITC. The resultant material was described as MCM-FITC.

KCC-FITC MSN

Amine-modified silica material (KCC-NH_2) was used in this study; first FTIC was reacted with APTES in separate reaction in dark condition, and then followed by using the KCC-NH_2 in order to save the attached amino groups on silica material to further modification or loading drug molecules. In typical preparation, at first, FITC (10 mg) was dissolved in absolute ethanol (7 ml), then APTES (100 µL) was added under stirring and the mixture was stirred for 2 h to obtain FITC-APTES solution. In separate flask, the 1 g of KCC-NH_2 was dispersed in absolute ethanol under stirring; after that the FITC-APTES solution dropped slowly into silica suspension and stirred for 24 h at room temperature. Finally, the material (yellow/orange product) was collected by centrifugation and washed with ethanol and dried at room temperature, the material was donated as KCC-FITC.

Folic acid-conjugation of MSNs

In the first step, in a flask containing 50 ml of DMSO, FA (10 mg) and APTES (10 µL) were added under stirring. Then, followed by adding N-hydroxysuccinimide (NHS, 3 mg) and 1-(3-dimethylaminopropyl)-3-ethylcarbodiimide hydrochloride (EDC, 5 mg) into the mixture and stirred for 2 hours at room temperature to obtain the "FA/APTES solution". In the second step, in a separate flask containing KCC-NH_2-DMSO or MCM-NH_2-DMSO suspension (1000 mg/50 ml) and toluene (30 mL), these suspensions were stirred for 2 h at room temperature. After that, the FA/APTES solution was added to silica-DMSO suspension and the mixture was stirred for 24 hours at room temperature. Finally, the both materials were recovered by centrifugation, washed several times

with anhydrous toluene and dried; the obtained materials were labeled as KCC-NH_2-FA and MCM-NH_2-FA material.

Physicochemical characterizations of the nano-materials

The structure of MSNs was observed by means of High Resolution Transmission Electron Microscope, HR-TEM (JEM 2100, JEOL, Japan). The morphology and chemical compositions of the samples were characterized by Field Emission Scanning Electron Microscope, FE-SEM (Ultra Plus, Zeiss, Germany) equipped with QUANTAX EDS (Bruker). The mesostrucure ordering of MSNs and loaded ones was characterized using powder X-ray diffraction, XRD (X´PertPRO System, PANalytical,) using CuKα radiation in the 2θ range of 10-100º. The mesoporosity characteristics (Brunauer-Emmett-Teller 'BET' surface area, pore size distributions and pore volume) were measured using (NOVA, Quantachrome Automated Gas Sorption SystemandGimin, Micromeritics, USA). In this context, all drug-loaded samples were degassed for 24 h at 50° C and the non-loaded samples degassed for shorter time and higher temp. (120° C for 12 h). The pore size distributions were obtained from the adsorption/desorption branches of the isotherms, based on the density functional theory (DFT). FTIR spectra were conducted using a Fourier transformed infrared (FTIR) spectroscopy (Bruker Optics Tensor 27, Bruker Corporation, Billerica, MA, USA) equipped with a Attenuated Total Reflectance (ATR, model Platinium ATR-Einheit A 255) to identify the surface functional groups. Simultaneous Thermal Analysis (STA)-coupled with Differential Scanning Calorimetry (DSC) (STA-DSC) analysis was performed using (STA 499 F1Jupiter, NETZSCH-Feinmahltechnik GmbH, Germany). This analysis was used for two reasons: firstly to determine the drug loading and efficiency by STA, and secondly to confirm the crystalline state by DSC. Samples weighting ca. 10–20 mg were loaded into alumina pan of the STA unit, and before measurements, helium was flown through the STA furnace chamber for 30 min. The experimental parameters were programmed to reach 800°C with a heating rate of 10° C/min under a helium/air mixture. The surface charges on surface of calcined, functionalized and drug-loaded MSNs were measured by zeta potential measurements using a Malvern ZetaSizer (NanoZS, UK); the measurements were performed based on water suspension of nanoparticles (concentration of 1 mg/ml) at 24° C, and also various pH values were investigated. The particle size distribution of all MSNs was characterized by nanoparticle tracking analysis (NTA) using NanoSight instrument (NS500, NanoSight, UK). The water suspensions of nanoparticles with concentration of 15 mg/20 ml were utilized during all measurements; the results were collated and analyzed by NanoSight software.

N2 adsorption/desorption measurements

As shown in Supplementary Table 1, N2 adsorption/desorption measurements showed that the surface area and total pore volume were reduced after drug loading to FA-conjugated MSNs (CR: MCM-NH_2-FA-CR, KCC-NH_2-FA-CR; QR: MCM-NH_2-FA-QR, KCC-NH_2-FA-QR; COL: MCM-NH_2-FA-COL and KCC-NH_2-FA-COL) compared to as-synthesized MSNs (MCM-Calcined and KCC-Calcined), indicating successful drug loading for FA-conjugated MSNs. After the surface modifications and drug loading processes, the mean size of nanoparticles slightly increased.

FT-IR results

The as-synthesized MSNs (MCM-Calcined and KCC-Calcined) (Figure 4A) showed many bands corresponding to the siliceous framework, including a band at 1070 cm^{-1}, 810 cm^{-1}, and 450 cm^{-1}. In case of FTIC fluorescent labeled to MSNs, the FT-IR spectra of MCM labeled with FITC fluorescent dye (MCM-FITC) displayed a new band at 960 cm^{-1}. This band did not appear in KCC labeled with FITC fluorescence (KCC-FITC) (Figure 4A) may be due to different synthesis methods. Further functionalization of as-synthesized MSNs with aminopropyl groups by APTES (MCM-NH_2 and KCC-NH_2) lead to the appearance of new bands in the range from 1350 cm^{-1} to 1700 cm^{-1} (Figure 4A and Supplementary Figure 2). In FA-conjugated MSNs, the bands at 1556 cm^{-1}, 1495 cm^{-1}, and 1390 cm^{-1} became weaker compared to amino-modified MSNs, and three new bands were observed at 1440 cm^{-1}, 1407 cm^{-1}, and 1315 cm^{-1}.

Zeta potential measurements

Zeta potential/pH curves for calcined, functionalized, dye-labeled FITC, and FA-conjugated MSNs are shown (Figure 5A). In case of calcined MSNs, at pH 2–2.5, both MCM-Calcined and KCC-Calcined MSNs showed zero values, while with further increase in the pH to 12, they acquired negative charges. In the case of dye-labeled MSNs, at low pH 2–2.5, they displayed positively charged values. With an increase of pH to 5.5, the MCM-FITC showed negative charges. KCC-FITC acquired negative charges for pH higher than 8.5. Furthermore, increasing pH to 12 resulted in both acquiring high negative charges. In case of amino-modified MSNs, both MCM-NH_2 and KCC-NH_2 possessed positive charges for pH values from 2 to 8. The KCC-NH_2 exhibited higher positive charges than MCM-NH_2. By increasing the pH to 12, they both showed negative charges.

Supplementary Table 1: Physicochemical properties of FA-conjugated MSNs and prodrugs loaded to FA-conjugated MSNs

Sample	S_{BET} (m^2/g)	Pore Volume[a] (cm^3/g)	Mean size Distribution[b] (nm)	Amount of FA[d] (wt.%)	Elemental analysis[e]		Loading Content (wt.%)[f]
					N%	C%	
**MCM-NH$_2$-FA**	34	0.195	233 ± 18	2.47	2.59	14.91	-
**KCC-NH$_2$-FA**	45	0.192	346 ± 11	3.93	3.39	16.96	-
**MCM-FA-CR**	4.5	0.168	248 ± 9	-	2.22	13.98	29.3
**KCC-FA-CR**	8	0.057	394 ± 20	-	3.10	16.36	19.5
**MCM-FA-QR**	16	0.132	245 ± 41	-	2.07	14.07	31.6
**KCC-FA-QR**	13.9	0.125	402 ± 9	-	3.14	15.87	20.3
**MCM-FA-COL**	25.7	0.574	314 ± 29	-	1.99	13.56	2.9
**KCC-FA-COL**	30	0.323	454 ± 18	-	3.14	15.87	2.3

[a]Pore volume from Nitrogen adsorption/desorption measurements.
[b]Mean size distribution from NanoSight measurement.
[c&d]Calculated from STA analysis.
[e]Obtained from EDS analysis.
[f]Calculated from STA analysis.

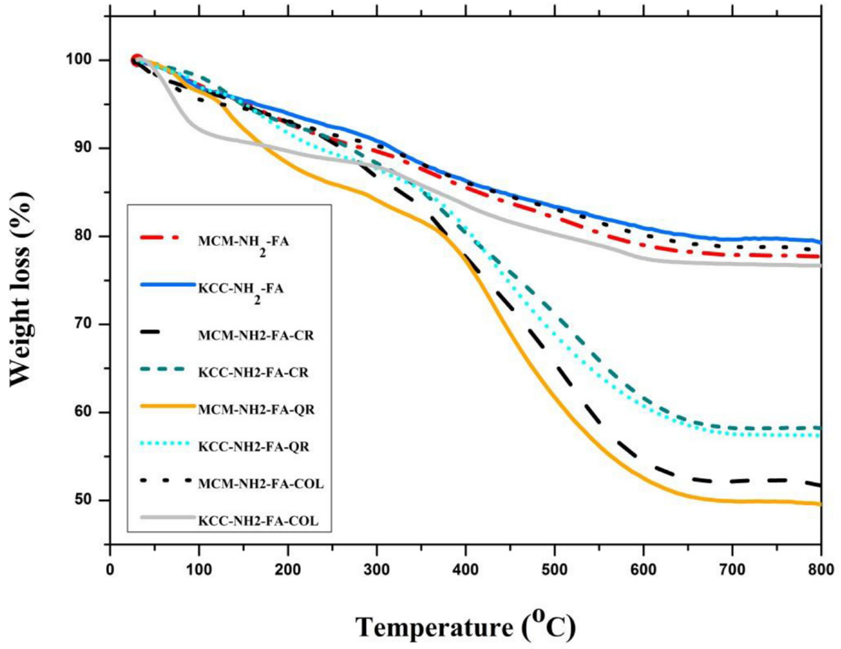

Supplementary Figure 1: STA profiles of the FA-conjugated to MSNs of both types, and prodrug-loaded FA-conjugated MSNs.

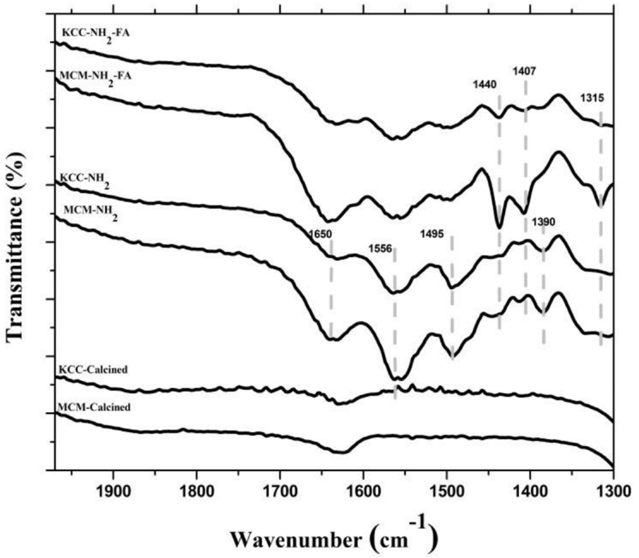

Supplementary Figure 2: FTIR spectra of amine-functionalized and FA-conjugated MSNs.
